# The cost and cost efficiency of conducting a 24-h dietary recall using INDDEX24, a mobile dietary assessment platform, compared with pen-and-paper interview in Viet Nam and Burkina Faso

**DOI:** 10.1017/S0007114522001362

**Published:** 2023-02-14

**Authors:** Katherine P. Adams, Winnie Bell, Jérome W. Somé, Brooke Colaiezzi, Sarah Wafa, Beatrice Rogers, Jennifer Coates

**Affiliations:** 1 Institute for Global Nutrition, University of California, Davis, CA, USA; 2 Gerald J. and Dorothy R. Friedman School of Nutrition Science and Policy, Tufts University, Medford, MA, USA; 3 Institut de Recherche en Sciences de la Santé, Bobo-Dioulasso, Burkina Faso

**Keywords:** 24-h dietary recall, Cost, Cost efficiency, Computer-assisted personal interview (CAPI), Pen-and-paper interview (PAPI)

## Abstract

The INDDEX24 Dietary Assessment Platform (INDDEX24) was developed to facilitate the collection of 24-h dietary recall (24HR) data. Alongside validation studies in Viet Nam and Burkina Faso in 2019–2020, we conducted activity-based costing studies to estimate the cost of conducting a 24HR among women of reproductive age using INDDEX24 compared with the pen-and-paper interview (PAPI) approach. We also modelled alternative scenarios in which: (1) 25–75 % of dietary reference data were borrowed from the INDDEX24 Global Food Matters Database (FMDB); (2) all study personnel were locally based and (3) national-scale surveys. In the primary analysis, in Viet Nam, the 24HR cost US $111 004 ($755/respondent, *n* 147) using INDDEX24 and $120 483 ($820/respondent, *n* 147) using PAPI. In Burkina Faso, the 24HR cost $78 105 ($539/respondent, *n* 145) using INDDEX24 and $79 465 ($544/respondent, *n* 146) using PAPI. In modelled scenarios, borrowing dietary reference data from the FMDB decreased the cost of INDDEX24 by 17–34 % (Viet Nam) and 5–15 % (Burkina Faso). With all locally based personnel, INDDEX24 cost more than PAPI ($498 *v*. $448 per respondent in Viet Nam and $456 *v*. $410 in Burkina Faso). However, at national scales (*n* 4376, Viet Nam; *n* 6500, Burkina Faso) using all locally based personnel, INDDEX24 was more cost-efficient ($109 *v*. $137 per respondent in Viet Nam and $123 *v*. $148 in Burkina Faso). In two countries and under most circumstances, INDDEX24 was less expensive than PAPI. Higher INDDEX24 survey preparation costs (including purchasing equipment) were more than offset by higher PAPI data entry, cleaning and processing costs. INDDEX24 may facilitate cost-efficient dietary data collection.

Quantitative, individual dietary data are a crucial source of information for quantifying food consumption and nutrient intake and for assessing the adequacy of intake. Commonly collected by 24-h dietary recall (24HR), individual-level dietary data provide essential input into evidence-based design, monitoring and evaluation of nutrition and nutrition-sensitive programmes and policies^(1,2)^. However, the complexities, cost and time burden associated with collecting, processing and analysing these data have discouraged their collection and use at large scale, particularly in low- and middle-income country (LMIC) settings^(3–5)^.

To facilitate the collection and use of individual dietary data in LMIC, the International Dietary Data Expansion (INDDEX) Project, led by Tufts University Friedman School of Nutrition Science and Policy, has developed, tested and deployed the INDDEX24 Dietary Assessment Platform (INDDEX24) to collect and analyse 24HR data. The novel platform, which links a web-based application that houses dietary reference data (e.g. food composition data, food and recipe listings, standard recipes, food descriptors, and portion conversion factors) to a mobile application (mobile app) for interviewer-based dietary data collection in the field on a smartphone or tablet, was developed with the intention of reducing both the complexity and resource requirements associated with traditional 24HR while maintaining or improving data quality.

Compared with other computer-assisted personal interview (CAPI) methods developed to collect 24HR data^(6–8)^, INDDEX24 has several unique features designed to ease some of the complexities and ultimately reduce the costs associated with preparing for a 24HR, conducting the survey and processing the data post data collection^(9)^ (online Supplementary Fig. S1). These features include the Global Food Matters Database (FMDB), which is an online, open-access database to store and organise the dietary reference data needed to collect and process dietary recall data, and an integrated analytical reports feature, which provides on-demand key summary statistics and a ‘gaps report,’ enabling researchers to quickly identify and update incomplete dietary reference data.

To our knowledge, the only relatively recent analysis of the cost of conducting 24HR in LMIC was done by Fiedler et al.^(5)^ using budget documents to estimate the cost of conducting a 24HR relative to a household consumption and expenditure survey. A number of other studies have assessed the cost of using CAPI relative to pen-and-paper interview (PAPI) for collecting health-related information, such as the cost of collecting health and demographic surveillance system data in Burkina Faso^(10)^, Malawi^(11)^ and Tanzania^(12)^. Other studies have compared the two modalities for the cost of collecting non-health-related data, such as data on the social impacts of conservation initiatives in Africa using CAPI *v*. PAPI^(13)^, or agricultural data in Tanzania and Uganda^(12)^. In general, these studies have found cost savings associated with collecting both health and non-health data using an electronic data collection system compared with PAPI, particularly for large-scale surveys. However, there is a need for more rigorous research on potential cost savings associated with electronic data collection generally^(14)^, and more specifically, there is insufficient evidence on the cost of conducting a 24HR in LMIC settings, including a gap in the evidence on the cost of conducting a 24HR using CAPI compared with PAPI modalities.

Conducted alongside validation studies of INDDEX24 in Viet Nam and Burkina Faso, we carried out activity-based costing studies in order to estimate and compare the cost of using INDDEX24 and the traditional PAPI modality to conduct a 24HR survey. This study adds to the sparse evidence on the cost of conducting 24HR in LMIC, and it fills the gap in knowledge on the cost of conducting 24HR using CAPI compared with PAPI modalities.

The specific objectives of this study were to: (1) assess the total and relative costs of conducting a 24HR and producing a clean, analysable 24HR dataset using INDDEX24 and using PAPI; (2) identify the sources of differences in costs between INDDEX24 and PAPI; (3) assess the cost efficiency (cost per respondent) of INDDEX24 compared with PAPI and (4) compare the time per respondent to complete the 24HR interview using INDDEX24 compared with PAPI. Reported separately, we also estimated and compared the cost-effectiveness of the two modalities based on measures of accuracy of 24HR data collected via INDDEX24 and via PAPI compared with a benchmark weighed food record (WFR)^(15)^.

The findings of this study provide researchers and decision-makers with detailed estimates of the cost of conducting 24HR, which can help with planning and budgeting for dietary data collection surveys of various scales. The results can also help inform their decisions about which modality of data collection to pursue based on cost and time per interview, and, with the results of the cost-effectiveness analyses, based on cost per unit of accuracy^(15)^.

## Subjects and methods

### Validation studies

The validation studies, described in detail ^(15,16)^, were conducted in 2019–2020 with women of reproductive age (18–49 years) drawn from rural households in the Thanh Oai District of Hanoi Province in the Red River Delta region in northern Viet Nam and rural households in the Plateau Central Region of Burkina Faso. In both countries, the 24HR were validated against a benchmark observer WFR by administering a WFR and, on the next day, a 24HR to the same women using either INDDEX24 or the traditional PAPI method to collect the data. In both countries, 234 women were recruited and randomly assigned to either the INDDEX24 arm or the PAPI arm (117 women per arm). In Viet Nam, dietary recall data were collected for all 234 women, while in Burkina Faso dietary recall data were observed for 115 women in the INDDEX24 arm and 116 women in the PAPI arm. The 24HR surveys were administered once per respondent (i.e. no repeat recall surveys were collected, because the primary study objectives were to compare accuracy of data collection modalities rather than to calculate usual intake with the widely validated 24HR method^(17)^).

In addition to the validation study samples, the 24HR surveys were also administered to sixty additional women in each country for whom the WFR was not collected on the day prior to the 24HR. Because this sample of women were selected from communities not exposed to the validation study, and the women did not undergo the WFR, this ‘naïve’ sample of women were included in order to record and compare the total time required to administer a 24HR survey using INDDEX24 (*n* 30) and PAPI (*n* 30). In each country, the sample of 60 respondents was evenly stratified across urban and rural study sites, and the data collection modality was randomised (fifteen women via INDDEX24 and fifteen women via PAPI in rural communities, and fifteen women via INDDEX24 and fifteen women via PAPI in urban communities). The cost study included both samples of women in the main study and the naïve sample, so total sample sizes for the cost study were 294 (*n* 147 In the INDDEX24 arm and *n* 147 in the PAPI arm) in Viet Nam and 291 (*n* 145 in the INDDEX24 arm and *n* 146 in the PAPI arm) in Burkina Faso.

The 24HR surveys administered to women in the validation studies and women in the ‘naïve’ samples used the multiple-pass 24-h interview method in which information on dietary intake is collected in four distinct ‘passes’. The 1st pass, also known as the ‘quick list’, is designed to collect a quick summary of all foods consumed in the previous 24 h; during the 2nd pass detailed information about each food consumed (e.g. cooking method, variety, and fat content) is gathered; in the 3rd pass, an estimate of the quantity consumed is obtained; and in the 4th pass, a review of all foods reported is conducted with the respondent for accuracy and completeness^(18)^. An additional pass to collect details on non-standard recipes is sometimes collected in cases where the mixed dish consumed diverges from the standard recipe in the database. The PAPI form was designed to be as similar as possible to the INDDEX24 mobile application and followed the same sequencing with the four passes and non-standard recipe details.

In Viet Nam, the validation and cost studies were conducted in collaboration with the National Institute of Nutrition (NIN), a division of the Ministry of Health. In Burkina Faso, the studies were conducted in collaboration with Institut National de la Statistique et de la Démographie (INSD), the national bureau of statistics at the Ministry of Economy and Finance.

### Cost studies

We conducted an activity- and ingredients-based costing study^(19)^ alongside the validation study in each country in order to measure the total and relative economic costs of conducting a 24HR survey and producing a clean and analysable 24HR dataset using the INDDEX24 Platform compared with PAPI. We defined and costed a series of activities (as well as subactivities/tasks) required to complete data collection and prepare the datasets, including preparation of dietary reference data, survey preparation, training, survey execution, data entry, and data cleaning and processing. Then, we identified the types and quantities of inputs, or ingredients, that were required to execute each activity. These ingredients included personnel, facilities, travel, transportation, lodging, per diem, equipment, supplies and other (IRB fees and overhead). We defined costs from a societal perspective in which all costs were included regardless of who incurred them. We also calculated costs from the perspective of the study participants. Supplementary Table S1 shows the primary activities and subactivities that were costed, separately, for the INDDEX24 and PAPI modalities in each country, including detailed breakdown of the components of each subactivity.

We developed a series of instruments to collect the time and monetary costs associated with completing each activity. Field staff recorded their daily time use for all field-based activities (i.e. training, sampling, conducting interviews, conducting data quality control, data entry, data cleaning and processing, and preliminary analysis) using a combination of paper-based quick logs to track time during the day and position-specific time logs entered at the end of each day via Google Forms. Prior to the start of data collection, field staff were trained on the use of the quick logs and time logs. This training included making field staff aware that their daily time use data would not be used as a tool to monitor their performance, but rather it would be used in an anonymous way by off-site researchers to calculate the time needed to conduct 24HR using INDDEX24 and PAPI.

During data collection, field staff time use data were reviewed by local supervisors, and errors or inconsistencies were identified and corrected where needed. INDDEX study staff and staff at NIN in Viet Nam and at INSD in Burkina Faso recorded their activity-specific time use and monetary expenditures using Excel-based time and expenditures reporting logs, which were differentiated by data collection modality (INDDEX24 and PAPI).

We used the salary or wage received by field-based personnel (enumerators, field supervisors, data entry clerks and data supervisors) and other in-country personnel (translators, coordinators, in-country researchers, technical advisors and chefs) to value the time of study staff based in Viet Nam and Burkina Faso. The time of US-based staff (project administrators, researchers and lead researchers/principal investigators – all of whom worked on the project from US except for a researcher on the Viet Nam validation study who spent several months in the field) was valued based on average salary estimates, adjusted to 2019 US dollars, for comparable positions at US research institutions according to published data from The Chronicle of Higher Education^(20)^. Because the data source for average salary estimates did not include salary information for statisticians at research universities, the value of the statistician’s time was based on the average salary of a mid-level biostatistician at Tufts University. Survey respondents’ time, which included total average time to conduct the 24HR module plus the respondents’ time required for recruitment and consent (about 15 min per respondent), was valued at the average of the region-specific minimum monthly wages of 3 151 000 Vietnamese dong (or about $151 US dollars)^(21)^ and at the minimum wage in Burkina Faso of 34 664 West African CFA franc (about $59) per month^(22)^.

All costs were adjusted to 2019 US dollars. For costs paid in US dollars, costs incurred in 2018 were adjusted to 2019 US dollars using the Bureau of Economic Analysis implicit price deflators for gross domestic product^(23)^. For costs paid in Vietnamese dong (Viet Nam) or West African CFA francs (Burkina Faso), costs were first adjusted to the 2019 value (where necessary) using the local GDP price deflator, then converted to US dollars using the average 2019 exchange rate.

Because most of the equipment used in the surveys had a useful life of longer than 1 year and can be used for future surveys, costs were annualised over the useful life of the item as described in Drummond, Sculpher^(24)^, using a 3 % discount rate. We used annualised costs for food scales (assuming a useful life of 2 years), tablets and computers (assuming a useful life of 3 years), portable hard drives (assuming a useful life of 4 years), and storage cabinets and standard weights (assuming a useful life of 10 years). In cases where equipment is not used beyond a single survey, the full cost of the equipment should be included. Also note that, due to risk of loss or damage and to ensure sufficient equipment during the survey period, several backup scales and tablets were purchased and included in the total equipment costs.

Finally, it is important to characterise the nature of the work done in each country to develop dietary reference data, as these differences impacted the cost of developing the dietary reference data in each country. In Viet Nam, the development of dietary reference data primarily occurred prior to data collection and was quite extensive, as it was done not only for the INDDEX24 validation study but also in preparation for the 2019–2020 national General Nutrition Survey. As such, the food list, standard recipes, conversion factors (including density factors), portion size estimation methods, photo atlas and other critical inputs were all developed with the intention of being relevant for diets across Viet Nam. The dietary reference data preparation work in Viet Nam also included adding many cooked foods to the published Vietnamese Food Composition Table^(25)^. In Burkina Faso, the work to develop the dietary reference data was focused only on the specific region in Burkina Faso where the validation study took place, and it also benefited from previous development of dietary reference data for that region that could, to a large extent, be borrowed from, including recipes, photos for the photo book, density factors, portion size estimation methods, food composition data and conversion factors.

### Scenarios

The validation studies were conducted under a specific set of circumstances that may not always reflect the circumstances under which a 24HR would be conducted. First, the database from which INDDEX24 draws dietary reference data, the FMDB, is currently in a very nascent stage. As users contribute new data to the FMDB (e.g. food composition tables, standard recipes, food descriptor lists and portion conversion factors), it is expected that the effort required to prepare dietary reference data for a 24HR will decrease, since users may be able to borrow from dietary reference data shared by previous users. Because data in the FMDB are primarily country-specific (or region-specific), the potential time-savings will increase as data from more countries are added so that each new survey can build on the data already available. Second, many of the people working on the validation study were internationally based, and we anticipate that groups using the INDDEX24 Platform will often be locally based and employ primarily local leadership and staff. And finally, the validation study sample sizes were small, and respondents were recruited from relatively small geographic areas within each country. We anticipate that users of INDDEX24 will often use the system to collect 24HR data at a larger scale. To assess the impact of differing assumptions about these factors on the cost and cost efficiency of INDDEX24 relative to PAPI, we estimated costs under a number of different modelled scenarios, as described in Table 1, relative to the primary analysis (i.e. the analysis that reflects the conditions under which costs were collected and analysed for the validations studies).

**Table 1.** Characteristics of cost scenarios relative to the primary cost analysis

		Sample size		Personnel		Dietary reference data	
Scenario	Country	INDDEX24	PAPI	INDDEX24	PAPI	INDDEX24	PAPI
Primary analysis	Viet Nam	147	147	Leadership positions* filled by US-based personnel		Nationally representative, comprehensive dietary reference database developed prior to data collection	Nationally representative, comprehensive dietary reference database developed prior to data collection
	Burkina Faso	145	146	Leadership positions (excluding researcher) filled by US-based personnel		Regionally representative, comprehensive dietary reference data database developed prior to data collection	Regionally representative, comprehensive dietary reference data database developed prior to data collection
Scenario 1: Borrow dietary inputs from FMDB	Viet Nam	Same as primary analysis		Same as primary analysis		25, 50 and 75 % borrowed from the FMDB	Same as primary analysis
	Burkina Faso	Same as primary analysis		Same as primary analysis		25, 50 and 75 % borrowed from existing dietary reference data	Same as primary analysis
Scenario 2: All in-country personnel	Viet Nam	Same as primary analysis		All positions filled by in-country personnel		Same as primary analysis	
	Burkina Faso	Same as primary analysis		All positions filled by in-country personnel		Same as primary analysis	
Scenario 3: National survey	Viet Nam	4376	4376	All positions filled by in-country personnel		Same as primary analysis	
	Burkina Faso	6500	6500	All positions filled by in-country personnel		Same as primary analysis	

INDDEX24, INDDEX24 Dietary Assessment Platform; PAPI, pen-and-paper interview; FMDB, Food Matters Database.

* Leadership positions include lead researcher/principal investigator, researcher, statistician and administrator.

As previously described, one of the goals of the INDDEX24 Platform is to provide a well-developed database of dietary reference data (via the FMDB) for many different countries from which future users of INDDEX24 can draw, thereby reducing the time and cost of preparing dietary reference data. As the validation studies in Viet Nam and Burkina Faso represented the first uses of INDDEX24, there were no pre-existing dietary reference data in the FMDB, so all dietary reference data had to be developed and/or reviewed and loaded into the INDDEX24 database. To model the potential impact on costs when future users are able to draw from existing reference data housed in the FMDB, we modelled a set of scenarios in which 25, 50 and 75 % of the dietary reference data needed to conduct a 24HR and analyse the data were already available in the FMDB (note that even if all dietary reference data are available in the FMDB, some work will be required to review and update the existing data as needed). For these scenarios, we assumed the cost associated with the preparation of dietary reference data would decrease proportionally (i.e. the cost of preparing dietary reference data would decrease by 25, 50 and 75 %).

For the all in-country personnel scenario, based on input from collaborators at NIN in Viet Nam and INSD in Burkina Faso, for each position that was filled by US-based personnel during the validation study (lead researcher/principal investigator, coordinating researcher, other researchers, statistician, and administrator in Viet Nam, and lead researcher/principal investigator, other researchers, statistician, and administrator in Burkina Faso), we identified an in-country equivalent position. All time costs were then recalculated based on in-country salary estimates of the equivalent positions. Because the coordinating researcher position in Burkina Faso was filled by a Burkinabe during the validation study, this position and salary were maintained for the in-country personnel scenario.

Finally, we modelled a scenario in which the 24HR surveys were assumed to be national in scale and all positions (including leadership positions) filled by in-country personnel. Modelling this scenario required the development of two main sets of assumptions. The first set of assumptions were related to the assumed sample sizes for national surveys in Viet Nam and Burkina Faso, the time frame for data collection, and the number of enumerators, supervisors, and data entry clerks, measured in full-time equivalents, that would be required to undertake the national surveys. The second set of assumptions were related to the nature of each cost component of each activity (i.e. fixed *v*. variable cost, and if variable, how costs would increase as the sample size increased).

We developed the sample size estimates for the national surveys based on the sample sizes used for the 2019–2020 General Nutrition Survey conducted by NIN in Viet Nam (representative nationally and of Viet Nam’s six agro-ecological zones) and planning for the upcoming Food Consumption Survey in Burkina Faso (representative nationally and of Burkina Faso’s thirteen regions). We assumed the national surveys would sample women of reproductive age and would be representative both nationally and subnationally. Table 2 below presents the sample size, time frame and full-time equivalents requirements upon which the national survey scenario models were based. Other underlying assumptions are summarised in Supplementary Table S2. Our calculations accounted for repeat 24HR surveys to be administered to a random subset of 20 % of women on non-consecutive days. We also assumed that in each country, there would be several subnational ‘hubs’ from which training and data collection would be coordinated. Each subnational hub was assumed to cover several regions/zones, and we assumed that data collection would occur simultaneously over a 1-month period in each of the regions/zones covered by each hub. Finally, we assumed that the national team would have the background, training and/or experience to plan, prepare for and execute the national 24HR without external training or support, so we did not include any time or expenses associated with capacity building. For countries requiring external support, the time and costs associated with capacity building should be included.

**Table 2.** National scenario sampling and data collection assumptions

	Viet Nam	Burkina Faso
Sample size		
Sample size for national survey of WRA	4376	6500
Sample size for national survey of WRA including 20 % replicate surveys	5251	7800
Number of representative regions or zones	6	13
Average sample size per region or zone	875	600
Number of subnational ‘hubs’	6	5
Average sample size per subnational hub	875	1560
Duration and intensity of data collection		
Target duration of data collection (d)	30	30
Assumed number of days of data collection per week	7	7
Number of working days per enumerator per 30 d of data collection	26	26
Assumed average number of surveys completed/d per enumerator	5	4
Average number of surveys completed per enumerator over data collection period	130	104
Enumerator and field supervisor requirements*		
Enumerator FTE required per subnational hub	7	16
Field coordinator FTE per subnational hub	2	2
Field supervisor FTE required per subnational hub†	2	3
Total enumerator FTE	42	75
Total field coordinator FTE	12	10
Total field supervisor FTE	12	15
Duration and intensity of data entry		
Target duration of data entry (d)	30	30
Assumed number of days of data entry per week	7	7
Number of working days per clerk per 30 d of data collection	25·7	25·7
Assumed average number of surveys entered/d per clerk	6	6
Average number of surveys entered per clerk over data entry period	156	156
Data entry clerk and data supervisor requirements*		
Total data entry clerk FTE	68	100
Total data supervisor FTE‡	2	3

WRA, women of reproductive age; FTE, full-time equivalents.

* Enumerator, supervisor and data entry clerk requirement estimates are rounded to the nearest whole number.

† Assuming 1:6 ratio of field supervisors to enumerators.

‡ Assuming 1:10 ratio of data entry clerks to data supervisors.

### Ethical approval

Ethical approvals for the validation and cost studies were obtained from the institutional review board at Tufts University and the institutional review board at the National Institute of Nutrition (Viet Nam) and the National Ethics Review Committee (Burkina Faso). All respondents provided informed consent prior to participation in the studies.

## Results

### Primary analysis

**Fig. 1.**
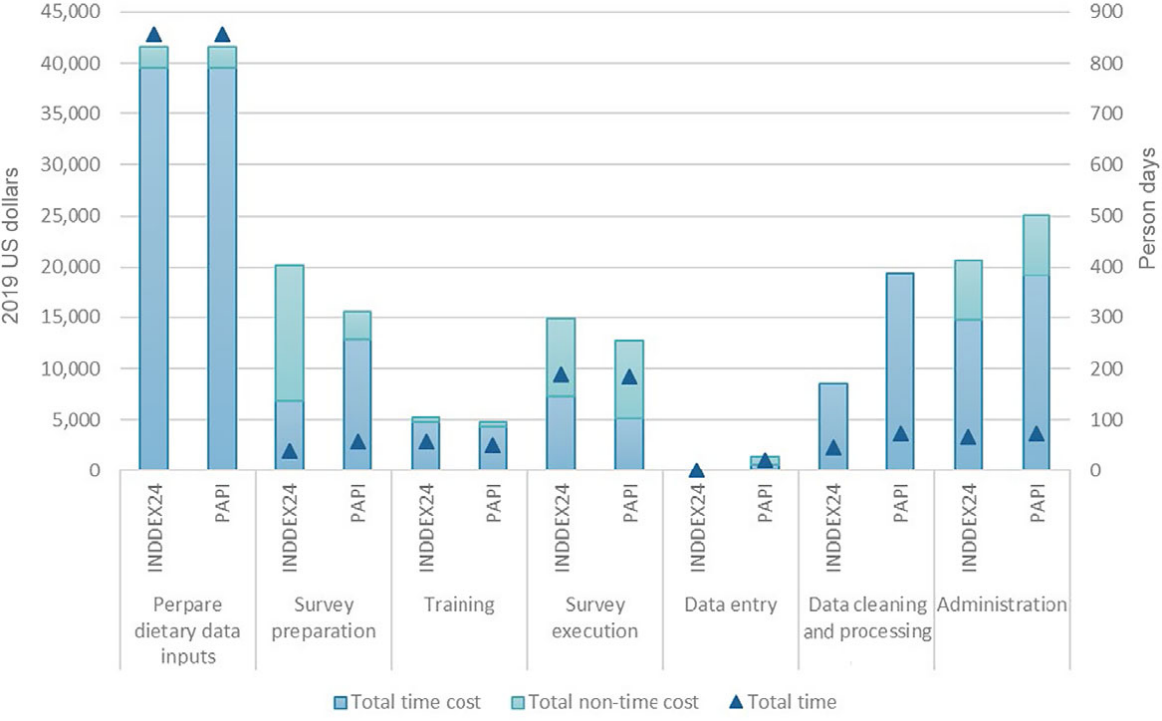
Time (human capital) and non-time (non-human capital) costs of conducting a 24-h dietary recall using INDDEX24 and PAPI by activity: Viet Nam. INDDEX24, INDDEX24 Dietary Assessment Platform; PAPI, pen-and-paper interview.

**Fig. 2.**
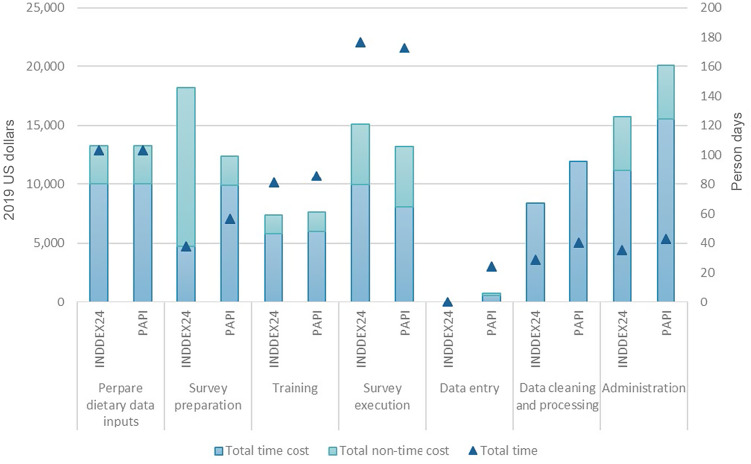
Time (human capital) and non-time (non-human capital) costs of conducting a 24-h dietary recall using INDDEX24 and PAPI by activity: Burkina Faso. INDDEX24, INDDEX24 Dietary Assessment Platform; PAPI, pen-and-paper interview

Table 3 presents the economic cost, from a societal perspective, of conducting the 24HR using INDDEX24 and using PAPI in Viet Nam. These cost estimates are disaggregated by time (human capital) and non-time (non-human capital) costs in Fig. 1. Table 4 and Fig. 2 present the analogous set of cost estimates for Burkina Faso.

**Table 3.** Economic cost and cost efficiency of conducting a 24HR using INDDEX24 and PAPI: Viet Nam

		INDDEX24		PAPI		Difference in cost*
Primary activity	Subactivities	Cost (2019 USD)	Percent of activity total	Cost (2019 USD)	Percent of activity total	INDDEX24-PAPI (2019 USD)
Preparation of dietary reference data	Develop food and recipe lists and tags/probes	14 098	33·9	14 098	33·9	0
	Prepare food composition table	1005	2·4	1005	2·4	0
	Develop standard recipes density factors	10 338	24·9	10 338	24·9	0
	Identify PSEM/conversion factors	12 452	30·0	12 452	30·0	0
	Compile and format dietary reference data	3659	8·8	3659	8·8	0
	Subtotal	41 552		41 552		0
Survey preparation	Design paper questionnaire for 24HR	0	0·0	2157	13·9	–2157
	Develop data entry form for 24HR	0	0·0	5423	34·9	–5423
	Pilot mobile app/paper questionnaires	2121	10·5	1118	7·2	1003
	Develop manuals and training materials	2285	11·3	1912	12·3	373
	Translate forms and training materials	1325	6·6	1325	8·5	0
	Print survey instruments/questionnaires	319	1·6	716	4·6	–397
	Print photo atlas	494	2·5	494	3·2	0
	Receive ethical approval	1590	7·9	1590	10·2	0
	Purchase and prepare supplies and equipment	2034	10·1	823	5·3	1211
	Purchase CommCare subscription	10 000	49·6	0	0·0	10 000
	Subtotal	20 168		15 557		4611
Training	Supervisor training†	0	0·0	0	0·0	0
	Enumerator training	5267	100·0	3779	79·4	1488
	Data entry clerk training	0	0·0	979	20·6	–979
	Subtotal	5267		4758		509
Survey execution	Household listing and sampling of eligible participants	2642	17·8	2642	20·7	0
	Incentives	1782	12·0	1782	14·0	0
	Data collection and field supervision	8327	56·1	8338	65·3	–11
	Electronic data monitoring	2089	14·1	0	0·0	2089
	Subtotal	14 840		12 762		2078
Data entry	Data entry and supervision	0	0·0	1405	100·0	–1405
	Subtotal	0		1405		–1405
Data cleaning, processing and preparation	Data cleaning, processing (food matching, gap filling, etc.) and preparation for analysis	8500	100·0	19 440	100·0	–10 939
	Subtotal	8500		19 440		–10 939
Administration	Management and oversight	14 795	71·6	19 127	76·5	–4333
	International travel to the field	1700	8·2	1700	6·8	0
	Lodging/per diem for international personnel	2027	9·8	2027	8·1	0
	Overhead	2156	10·4	2156	8·6	0
	Subtotal	20 677		25 009		–4333
Totals	Prepare dietary reference data	41 552	37·4	41 552	34·5	0
	Survey preparation	20 168	18·2	15 557	12·9	4611
	Training	5267	4·7	4758	3·9	509
	Survey execution	14 840	13·4	12 762	10·6	2078
	Data entry	0	0·0	1405	1·2	–1405
	Data cleaning, processing and preparation	8500	7·7	19 440	16·1	–10 939
	Administration	20 677	18·6	25 009	20·8	–4333
	Grand total	111 004	100·0	120 483	100·0	–9479
	Number of respondents	147		147		0
	Total per respondent	755		820		–64

24HR, 24-h dietary recall; INDDEX24, INDDEX24 Dietary Assessment Platform; PAPI, pen-and-paper interview; PSEM, portion size estimation method; USD, US dollars.

* The difference is calculated as the cost of INDDEX24 minus the cost of PAPI.

† Due to time constraints, supervisor training did not take place as a separate activity in Viet Nam.

**Table 4.** Economic cost and cost efficiency of conducting a 24HR using INDDEX24 and PAPI: Burkina Faso

		INDDEX24		PAPI		Difference in cost*
Primary activity	Subactivities	Cost (2019 USD)	Percent of activity total	Cost (2019 USD)	Percent of activity total	INDDEX24-PAPI (2019 USD)
Preparation of dietary reference data	Develop food and recipe lists and tags/probes	2112	15·9	2112	15·9	0
	Prepare food composition table	0	0·0	0	0·0	0
	Develop standard recipes density factors	7513	56·6	7513	56·6	0
	Identify PSEM/conversion factors	2066	15·6	2066	15·6	0
	Compile and format dietary reference data	1587	12·0	1587	12·0	0
	Subtotal	13 278		13 278		0
Survey preparation	Design paper questionnaire for 24HR	0	0·0	963	7·8	–963
	Develop data entry form for 24HR	0	0·0	4525	36·5	–4525
	Pilot mobile app/paper questionnaires	588	3·2	567	4·6	21
	Develop manuals and training materials	893	4·9	893	7·2	0
	Translate forms and training materials	1973	10·8	1924	15·5	49
	Print survey instruments/questionnaires	36	0·2	224	1·8	–188
	Print photo atlas	363	2·0	363	2·9	0
	Receive ethical approval	2062	11·3	2062	16·6	0
	Purchase and prepare supplies and equipment	2282	12·5	893	7·2	1389
	Purchase CommCare subscription	10 000	55·0	0	0·0	10 000
	Subtotal	18 197		12 413		5784
Training	Supervisor training	3069	41·7	3069	40·0	0
	Enumerator training	4297	58·3	4226	55·1	71
	Data entry clerk training	0	0·0	369	4·8	–369
	Subtotal	7366		7664		–298
Survey execution	Household listing and sampling of eligible participants	3626	24·0	3626	27·4	0
	Incentives	1124	7·4	1124	8·5	0
	Data collection and field supervision	8431	55·7	8434	63·7	–3
	Electronic data monitoring	1943	12·9	55	0·4	1889
	Subtotal	15 124		13 238		1886
Data entry	Data entry and supervision	0	0·0	768	100·0	–768
	Subtotal	0		768		–768
Data cleaning, processing and preparation	Data cleaning, processing (food matching, gap filling, etc.) and preparation for analysis	8370	100·0	12 001	100·0	–3632
	Subtotal	8370		12 001		–3632
Administration	Management and oversight	11 194	71·0	15 526	77·2	–4333
	International travel to the field	0	0·0	0	0·0	0
	Lodging/per diem for international personnel	0	0·0	0	0·0	0
	Overhead	4576	29·0	4576	22·8	0
	Subtotal	15 770		20 103		–4333
Totals	Prepare dietary reference data	13 278	17·0	13 278	16·7	0
	Survey preparation	18 197	23·3	12 413	15·6	5784
	Training	7366	9·4	7664	9·6	–298
	Survey execution	15 124	19·4	13 238	16·7	1886
	Data entry	0	0·0	768	1·0	–768
	Data cleaning, processing and preparation	8370	10·7	12 001	15·1	–3632
	Administration	15 770	20·2	20 103	25·3	–4333
	Grand total	78 105	100·0	79 465	100·0	–1360
	Number of respondents	145		146		–1
	Total per respondent	539		544		–6

24HR, 24-h dietary recall; INDDEX24, INDDEX24 Dietary Assessment Platform; PAPI, pen-and-paper interview; PSEM, portion size estimation method; USD, US dollars.

* The difference is calculated as the cost of INDDEX24 minus the cost of PAPI.

In Viet Nam, the total economic cost of the 24HR up to the point of producing a clean and analysable 24HR dataset was $111 004 using the INDDEX24 Platform and $120 483 using the PAPI modality. On a cost efficiency basis, the total cost per respondent was $755 (*n* 147) using the INDDEX24 Platform and $820 per respondent (*n* 147) using the PAPI modality. The preparation of dietary reference data, which were used by both the INDDEX24 Platform and the PAPI modality, represented the largest share of the cost for undertaking the 24HR using both INDDEX24 (about 37 %) and PAPI (about 34 %). As shown in Fig. 1 (numbers available in online Supplementary Table S3), the preparation of dietary reference data in Viet Nam required substantially more time (measured in person days) than any other activity and, ultimately, the most resources of any activity.

In Burkina Faso, the total economic cost of the 24HR up to the point of producing a clean and analysable 24HR dataset was $78 105 using the INDDEX24 Platform and $79 456 using the PAPI modality. The total cost per respondent was $539 (*n* 145) using INDDEX24 Platform and $544 (*n* 146) using PAPI. Survey preparation (including developing manuals and training materials, conducting training, and purchasing supplies and equipment) and administrative costs (including management and oversight as well as overhead) represented the largest shares of the total cost of the 24HR using INDDEX24 (about 23 % and about 20 % of the total cost, respectively), while administrative costs represented the largest share of total costs for the PAPI modality (about 25 %). As shown in Fig. 2 (numbers available in online Supplementary Table S4), in Burkina Faso, survey administration was the most time-intensive activity for both modalities.

In both countries, the INDDEX24 Platform had higher costs than PAPI associated with survey preparation (which included the purchase of tablets and a 12-month subscription to CommCare, an open-source mobile platform, required for using the INDDEX24 mobile app) and survey execution. However, the cost savings associated with the INDDEX24 Platform for data entry, cleaning and processing/preparation for analysis as well as project administration more than offset the higher costs of survey preparation and execution relative to the PAPI modality. With costs disaggregated by cost centre, in both countries and for both the INDDEX24 and PAPI modalities, the largest share of costs (between 67 % and 83 %) were personnel costs, with personnel costs for the PAPI modality exceeding personnel costs for INDDEX24 in both countries (see online Supplementary Fig. S2 and S3).

Table 5 presents a comparison of the average time per respondent, overall and by pass, to conduct the 24HR module with the naïve samples of WRA in each country. In Viet Nam, the total time spent collecting data for the 24HR module was, on average, approximately 5 min faster using INDDEX24 (about 39 min, sd 15) compared with PAPI (about 44 min, sd 10), though the difference was not statistically significant. In Burkina Faso, completing the 24HR module was also slightly faster using the INDDEX24 (about 47 min, sd 18) modality compared with PAPI (about 50 min, sd 20), although again the difference was not statistically significant. When disaggregated by site, the total time difference between INDDEX24 and PAPI was slightly larger (and statistically significant at the 10 % level) among rural respondents compared with urban respondents in both countries. Apart from the non-standard recipes pass (both countries) and first pass (Burkina Faso only), the time to complete each pass was statistically significantly lower, at the 5 % level, using INDDEX24 than PAPI. Enumerators using INDDEX24 spent an average of 10 min (Viet Nam) or 18 min (Burkina Faso) per interview in the menu screen, which was not applicable for the PAPI interviews.

**Table 5.** Average time in minutes per 24HR among ‘naïve’* respondents

Country	Site	Modality	Total time per respondent		1st Pass†		2nd Pass‡		3rd Pass§		Non-standard recipe pass||		4th Pass¶		Menus**	
Viet Nam	Urban	INDDEX24 (*n* 15)	38·5	17·3††	6·2	2·0	7·7	2·9	10·7	5·8	2·7	5·1	1·4	0·7	9·8	4·6
		PAPI (*n* 15)	42·2	12·7	7·1	2·7	12·7	4·7	16·7	5·4	3·5	5·5	2·1	1·3	N/A	
		*P*-value‡‡	0·507		0·315		0·001		0·001		0·68		0·052			
	Rural	INDDEX24 (*n* 15)	39·6	12·6	7·0	2·4	8·0	2·7	11·7	4·6	1·6	2·8	1·1	0·4	10·1	4·3
		PAPI (*n* 15)	46·1	6·9	9·1	3·7	14·1	3·0	20·5	5·8	0·7	1·9	1·8	0·7	N/A	
		*P*-value	0·088		0·079		0·000		0·000		0·317		0·003			
	Combined	INDDEX24 (*n* 30)	39·0	14·9	6·6	2·2	7·8	2·7	11·2	5·2	2·2	4·1	1·3	0·6	10·0	4·4
		PAPI (*n* 30)	44·2	10·3	8·1	3·4	13·4	3·9	18·6	5·8	2·1	4·3	2·0	1·0	N/A	
		*P*-value	0·125		0·047		0·000		0·000		0·964		0·002			
Burkina Faso	Urban	INDDEX24 (*n* 15)	57·7	15·3*	9·8	3·1	8·4	3·3	11·3	4·2	26·4	8·3	3·5	1·8	21·6	9·7
		PAPI (*n* 15)	55·0	22·3	8·6	2·7	10·4	4·0	14·8	9·3	25·0	13·1	1·1	0·6	N/A	
		*P*-value‡	0·705		0·281		0·171		0·202		0·747		0·0001			
	Rural	INDDEX24 (*n* 15)	35·5	11·6	7·5	1·8	7·0	2·2	10·0	3·6	17·1	7·9	2·5	0·9	14·8	9·3
		PAPI (*n* 15)	45·2	17·7	9·8	3·6	8·9	3·1	13·0	4·6	14·3	10·2	1·0	0·4	N/A	
		*P*-value	0·093		0·043		0·061		0·062		0·542		0·000			
	Combined	INDDEX24 (*n* 30)	47·0	17·5	8·7	2·8	7·7	2·9	10·7	3·9	23·3	9·1	3·0	1·5	18·3	10·0
		PAPI (*n* 30)	49·9	20·3	9·2	3·2	9·6	3·6	13·8	7·2	19·4	12·6	1·1	0·5	N/A	
		*P*-value	0·552		0·515		0·031		0·041		0·254		0·000			

24HR, 24-h dietary recall; INDDEX24, INDDEX24 Dietary Assessment Platform; PAPI, pen-and-paper interview.

* The 24HR surveys were administered to sixty additional women in each country for whom the weighed food record was not collected on the day prior to the 24HR. Because this sample of women were selected from communities not exposed to the validation study, and the women did not undergo the WFR, this ‘naïve’ sample of women were included in order to record and compare the total time required to administer a 24HR survey using INDDEX24 and PAPI.

† The ‘1st Pass’ refers to the first stage of the 24HR when a ‘quick list’ of all foods consumed in the past 24 h is collected from the participant.

‡ The ‘2nd Pass’ refers to the second stage of the 24HR when detailed information on each food is recorded.

§ The ‘3rd Pass’ refers to the third stage of the 24HR when an estimate of the quantity consumed is collected.

|| The ‘Non-Standard Recipe Pass’ occurs in 24HR when the respondent reports a mixed dish that diverges from the standard recipes in which case detailed information on the amount prepared and ingredients used is recorded.

¶ The ‘4th Pass’ refers to the final stage of the 24HR when all items and quantities reported are reviewed by the enumerator and checked with the participant.

** The ‘Menu’ refers to the home screen in the INDDEX24 mobile app, which the enumerator must return to in between each pass of the 24HR. This is not relevant when conducting 24HR with PAPI.

†† Values are means (standard deviations).

‡‡ *P*-values for *t* test of difference in means between INDDEX24 and PAPI.

Based on the total average time to conduct the 24HR module plus the respondents’ time required for recruitment and consent (about 15 min per respondent), in Viet Nam, the average cost of participation in the 24HR survey was $0·81 per respondent in the INDDEX24 arm and $0·89 per respondent in the PAPI arm. In Burkina Faso, the average cost of participation was $0·36 in the INDDEX24 arm and $0·38 in the PAPI arm, with country differences reflecting different minimum wage rates in the two countries.

### Scenario 1: borrow from Food Matters Database

Under the scenarios in which dietary reference data were assumed to be borrowed from the FMDB for the INDDEX24 modality, the cost of conducting a 24HR using INDDEX24 decreased relative to using the PAPI modality (Table 6). In Viet Nam, the cost per respondent was predicted to drop by about 17–34 % if between 25 and 75 % of dietary reference data were borrowed from the FMDB, resulting in a savings of $136 to $277 per respondent using the INDDEX24 Platform compared with using the PAPI modality. In Burkina Faso, borrowing from the FMDB was predicted to decrease the cost per respondent by about 5–14 %, resulting in a savings of $28 to $74 per respondent using the INDDEX24 compared with PAPI.

**Table 6.** Economic cost and cost efficiency of conducting a 24-h dietary recall using INDDEX24 and PAPI assuming 25, 50 and 75 % of dietary reference data borrowed from the Global Food Matters Database

			INDDEX24		
Country	Primary activity	PAPI (2019 USD)	25 % (2019 USD)	50 % (2019 USD)	75 % (2019 USD)
Viet Nam	Prepare dietary reference data	41 552	31 164	20 776	10 388
	Survey preparation	15 557	20 168	20 168	20 168
	Training	4758	5267	5267	5267
	Survey execution	12 762	14 840	14 840	14 840
	Data entry	1405	0	0	0
	Data cleaning, processing and preparation	19 440	8500	8500	8500
	Administration	25 009	20 677	20 677	20 677
	Grand total	120 483	100 616	90 228	79 841
	Number of respondents	147	147	147	147
	Total per respondent	820	684	614	543
	Difference in cost per respondent		–136	–206	–277
Burkina Faso	Prepare dietary reference data	13 278	9959	6639	3320
	Survey preparation	12 413	18 197	18 197	18 197
	Training	7664	7366	7366	7366
	Survey execution	13 238	15 124	15 124	15 124
	Data entry	768	0	0	0
	Data cleaning, processing and preparation	12 001	8370	8370	8370
	Administration	20 103	15 770	15 770	15 770
	Grand total	79 465	74 785	71 466	68 146
	Number of respondents	146	145	145	145
	Total per respondent	544	516	493	470
	Difference in cost per respondent		–28	–51	–74

INDDEX24, INDDEX24 Dietary Assessment Platform; PAPI, pen-and-paper interview; USD, US dollars.

Costs presented in 2019 USD.

### Scenario 2: all in-country personnel scenario

Table 7 presents summary cost estimates for conducting the 24HR surveys and preparing clean, analysable datasets assuming that all positions (both field staff and leadership positions) were filled by in-country personnel in Viet Nam and Burkina Faso (disaggregated by subactivity in Supplementary Tables S5 and S6). In both Viet Nam and Burkina Faso, utilising all in-country personnel was predicted to decrease the total cost of conducting the 24HR more using the PAPI modality than the INDDEX24 Platform, resulting in lower total costs using PAPI than INDDEX24. In Viet Nam, the total cost was estimated to decrease from $755 to $498 per respondent using INDDEX24 (a 34 % decrease) and from $820 to $448 using the PAPI modality (a 45 % decrease). In Burkina Faso, employing all local-based personnel resulted in an estimated decrease in the cost of the 24HR from $539 to $456 (15 % decrease) using INDDEX24 and from $544 to $410 (25 % decrease) using the PAPI modality.

**Table 7.** Economic cost and cost efficiency of conducting a 24-h dietary recall using INDDEX24 and PAPI assuming all in-country personnel

		INDDEX24		PAPI		Difference in cost
Country	Primary activity	Cost (2019 USD)	Percent of total	Cost (2019 USD)	Percent of total	INDDEX24-PAPI (2019 USD)
Viet Nam	Prepare dietary reference data	27 882	38·1	27 882	42·4	0
	Survey preparation	15 841	21·6	6034	9·2	9807
	Training	3297	4·5	2788	4·2	509
	Survey execution	13 143	18·0	12 762	19·4	381
	Data entry	0	0·0	1405	2·1	–1405
	Data cleaning, processing and preparation	2187	3·0	3327	5·1	–1140
	Administration	10 861	14·8	11 614	17·6	–753
	Grand total	73 211	100·0	65 812	100·0	7399
	Number of respondents	147		147		0
	Total per respondent	498		448		50
Burkina Faso	Prepare dietary reference data	12 847	19·4	12 847	21·4	0
	Survey preparation	17 313	26·2	7923	13·2	9390
	Training	7366	11·1	7664	12·8	–298
	Survey execution	13 916	21·1	13 238	22·1	678
	Data entry	0	0·0	768	1·3	–768
	Data cleaning, processing and preparation	3437	5·2	4690	7·8	–1253
	Administration	11 184	16·9	12 766	21·3	–1581
	Grand total	66 063	100·0	59 895	100·0	6168
	Number of respondents	145		146		–1
	Total per respondent	456		410		45

INDDEX24, INDDEX24 Dietary Assessment Platform; PAPI, pen-and-paper interview; USD, US dollars.

### Scenario 3: national survey scenario

The modelled costs of conducting national-scale 24HR using INDDEX24 and PAPI are presented in Tables 8 and 9 for Viet Nam and Burkina Faso, respectively. We estimated that conducting a 24HR with a sample size of 4367 women of reproductive age (with 20 % replicate surveys) in Viet Nam would cost $477 267 using the INDDEX24 Platform and $601 001 using the PAPI modality, or $109 per respondent using INDDEX24 compared with $137 per respondent using PAPI. In Burkina Faso, the estimated total cost of conducting a national 24HR with 6500 women (20 % replicate surveys) was $802 385 using INDDEX24 ($123 per respondent) and $962 297 using PAPI ($148 per respondent). In both countries, although using the INDDEX24 platform was estimated to cost more than PAPI for survey preparation (including purchasing equipment) and survey execution (since using INDDEX24 allows for ongoing electronic data monitoring during data collection), the INDDEX24 cost savings of about $25 per respondent compared with PAPI was primarily due to substantially lower personnel requirements for data entry and data cleaning, processing, and preparation for analysis using INDDEX24 at national scale (see Supplementary Tables S7 and S8).

**Table 8.** Economic cost and cost efficiency of conducting a national-scale 24HR using INDDEX24 and PAPI: Viet Nam

		INDDEX24		PAPI		Difference in cost*
Primary activity	Subactivities	Cost (2019 USD)	Percent of activity total	Cost (2019 USD)	Percent of activity total	INDDEX24-PAPI (2019 USD)
Preparation of dietary reference data	Develop food and recipe lists and tags/probes	7400	26·5	7400	26·5	0
	Prepare food composition table	1005	3·6	1005	3·6	0
	Develop standard recipes density factors	10 338	37·1	10 338	37·1	0
	Identify PSEM/conversion factors	7299	26·2	7299	26·2	0
	Compile and format dietary reference data	1840	6·6	1840	6·6	0
	Subtotal	27 882		27 882		0
Survey preparation	Design paper questionnaire for 24HR	0	0·0	400	0·8	–400
	Develop data entry form for 24HR	0	0·0	518	1·0	–518
	Pilot mobile app/paper questionnaires	4169	7·7	4064	8·1	105
	Develop manuals and training materials	70	0·1	359	0·7	–289
	Translate forms and training materials	1325	2·4	1325	2·7	0
	Print survey instruments/questionnaires	11 388	21·0	25 564	51·2	–14 176
	Print photo atlas	5191	9·6	5191	10·4	0
	Receive ethical approval	963	1·8	963	1·9	0
	Purchase and prepare supplies and equipment	26 006	48·1	11 516	23·1	14 490
	Purchase CommCare subscription	5000	9·2	0	0·0	5000
	Subtotal	54 111		49 900		4212
Training	Supervisor training	21 892	33·1	21 892	31·0	0
	Enumerator training	44 339	66·9	44 339	62·9	0
	Data entry clerk training	0	0·0	4288	6·1	–4288
	Subtotal	66 231		70 519		–4288
Survey execution	Household listing and sampling of eligible participants	87 621	38·0	87 621	40·3	0
	Incentives	0	0·0	0	0·0	0
	Data collection and field supervision	129 223	56·0	129 632	59·7	–409
	Electronic data monitoring	14 003	6·1	0	0·0	14 003
	Subtotal	230 846		217 253		13 594
Data entry	Data entry and supervision	0	0·0	52 918	100·0	–52 918
	Subtotal	0		52 918		–52 918
Data cleaning, processing and preparation	Data cleaning, processing (food matching, gap filling, etc.) and preparation for analysis	24 972	100·0	98 057	100·0	–73 085
	Subtotal	24 972		98 057		–73 085
Administration	Management and oversight	26 824	36·6	26 824	31·8	0
	International travel to the field	0	0·0	0	0·0	0
	Lodging/per diem for international personnel	0	0·0	0	0·0	0
	Overhead	46 401	63·4	57 650	68·2	–11 249
	Subtotal	73 225		84 474		–11 249
Totals	Prepare dietary reference data	27 882	5·8	27 882	4·6	0
	Survey preparation	54 111	11·3	49 900	8·3	4212
	Training	66 231	13·9	70 519	11·7	–4288
	Survey execution	230 846	48·4	217 253	36·1	13 594
	Data entry	0	0·0	52 918	8·8	–52 918
	Data cleaning, processing and preparation	24 972	5·2	98 057	16·3	–73 085
	Administration	73 225	15·3	84 474	14·1	–11 249
	Grand total	477 267	100·0	601 001	100·0	–123 734
	Number of respondents	4376		4376		
	Total per respondent	109		137		–28

24HR, 24-h dietary recall; INDDEX24, INDDEX24 Dietary Assessment Platform; PAPI, pen-and-paper interview; PSEM, portion size estimation method; USD, US dollars.

* The difference is calculated as the cost of INDDEX24 minus the cost of PAPI.

**Table 9.** Economic cost and cost efficiency of conducting a national-scale 24HR using INDDEX24 and PAPI: Burkina Faso

		INDDEX24		PAPI		Difference in cost*
Primary activity	Subactivities	Cost (2019 USD)	Percent of activity total	Cost (2019 USD)	Percent of activity total	INDDEX24-PAPI (2019 USD)
Preparation of dietary reference data	Develop food and recipe lists and tags/probes	2957	16·4	2957	16·4	0
	Prepare food composition table	0	0·0	0	0·0	0
	Develop standard recipes density factors	10 518	58·5	10 518	58·5	0
	Identify PSEM/conversion factors	2893	16·1	2893	16·1	0
	Compile and format dietary reference data	1619	9·0	1619	9·0	0
	Subtotal	17 986		17 986		0
Survey preparation	Design paper questionnaire for 24HR	0	0·0	963	1·6	–963
	Develop data entry form for 24HR	0	0·0	657	1·1	–657
	Pilot mobile app/paper questionnaires	6344	7·9	6240	10·6	104
	Develop manuals and training materials	175	0·2	893	1·5	–718
	Translate forms and training materials	5825	7·2	5772	9·8	53
	Print survey instruments/questionnaires	1944	2·4	11 970	20·3	–10 025
	Print photo atlas	9528	11·8	9528	16·2	0
	Receive ethical approval	2011	2·5	2011	3·4	0
	Purchase and prepare supplies and equipment	49 784	61·8	20 821	35·4	28 964
	Purchase CommCare subscription	5000	6·2	0	0·0	5000
	Subtotal	80 612		58 853		21 758
Training	Supervisor training	27 205	24·2	27 205	23·5	0
	Enumerator training	85 013	75·8	85 013	73·6	0
	Data entry clerk training	0	0·0	3363	2·9	–3363
	Subtotal	112 218		115 580		–3363
Survey execution	Household listing and sampling of eligible participants	164 107	38·8	164 111	42·8	–4
	Incentives	0	0·0	0	0·0	0
	Data collection and field supervision	219 466	51·9	219 466	57·2	0
	Electronic data monitoring	39 007	9·2	0	0·0	39 007
	Subtotal	422 580		383 576		39 003
Data entry	Data entry and supervision	0	0·0	57 896	100·0	–57 896
	Subtotal	0		57 896		–57 896
Data cleaning, processing and preparation	Data cleaning, processing (food matching, gap filling, etc.) and preparation for analysis	49 207	100·0	193 126	100·0	–143 919
	Subtotal	49 207		193 126		–143 919
Administration	Management and oversight	46 046	38·4	47 100	34·8	–1054
	International travel to the field	0	0·0	0	0·0	0
	Lodging/per diem for international personnel	0	0·0	0	0·0	0
	Overhead	73 736	61·6	88 178	65·2	–14 442
	Subtotal	119 783		135 278		–15 496
Totals	Prepare dietary reference data	17 986	2·2	17 986	1·9	0
	Survey preparation	80 612	10·0	58 853	6·1	21 758
	Training	112 218	14·0	115 580	12·0	–3363
	Survey execution	422 580	52·7	383 576	39·9	39 003
	Data entry	0	0·0	57 896	6·0	–57 896
	Data cleaning, processing and preparation	49 207	6·1	193 126	20·1	–143 919
	Administration	119 783	14·9	135 278	14·1	–15 496
	Grand total	802 385	100·0	962 297	100·0	–159 912
	Number of respondents	6500		6500		
	Total per respondent	123		148		–25

24HR, 24-h dietary recall; INDDEX24, INDDEX24 Dietary Assessment Platform; PAPI, pen-and-paper interview; PSEM, portion size estimation method; USD, US dollars.

* The difference is calculated as the cost of INDDEX24 minus the cost of PAPI.

## Discussion

INDDEX24 was developed with the aim of making the collection, processing and analysis of 24HR data standardised and less resource-intensive. Alongside validation studies of the INDDEX24 Platform conducted in Viet Nam and Burkina Faso, we assessed the cost and cost efficiency of conducting a 24HR up to the point of producing a clean, analysable dataset using the INDDEX24 Platform and using the traditional PAPI modality. We found that from a societal perspective under the circumstance of the validation studies, using the INDDEX24 Platform cost $64 less per respondent in Viet Nam and $6 less per respondent in Burkina Faso compared with using the PAPI modality. Although the INDDEX24 Platform had higher costs associated with survey preparation, including the purchase of tablets and the fixed cost of purchasing a CommCare subscription, the relative overall cost savings in both countries were primarily derived from the lower cost of data entry, data cleaning and processing, and project administration when using INDDEX24 relative to PAPI.

From the perspective of respondents, we found that the time required to administer the dietary recall module was slightly lower, though not statistically significantly so, using the INDDEX24 Platform compared with PAPI in both Viet Nam (on average 39 min using INDDEX24 and 44 min using PAPI) and Burkina Faso (on average 47 min using INDDEX24 and 50 min using PAPI). Because we limited the assessment of respondent time costs to recruitment, consent and the administration of the dietary recall module, these cost estimates represent a lower bound on the cost respondents would face if additional survey modules (e.g. household demographics and socio-economic characteristics) or data collection activities (e.g. anthropometry) were conducted.

To provide information on the cost of conducting a 24HR under circumstances different from the validation studies, we also estimated costs under a set of alternative scenarios. Under each of these scenarios except the scenario assuming all personnel were locally based in Viet Nam and Burkina Faso, the predicted cost savings of using INDDEX24 relative to PAPI increased compared with the validation study-based estimates. The ability to borrow dietary reference data from the FMDB in the future represents a possibility for considerably lowering the relative cost of using the INDDEX24 Platform, particularly in contexts where the preparation of dietary reference data would otherwise be very time- and cost-intensive (i.e. contexts in which there is little pre-existing dietary reference data to draw from and where dietary patterns and foodways are complex and heterogeneous). In Viet Nam, where the preparation of dietary reference data was extensive (developed with the intention of being relevant for diets across Viet Nam, not just in the validation study area), we predicted that borrowing between 25 and 75 % of dietary reference data from the FMDB would reduce overall costs by 17–34 %. In Burkina Faso, where the preparation of dietary reference data was less extensive because it was focused on one small geographic region for which dietary reference data had previously been developed and could, to a large extent, be borrowed from, we predicted that overall costs might decrease by 5–14 %. Of course, 24HR surveys being implemented using the PAPI modality will also be able to access the dietary reference data contained in the Global FMDB, but using those dietary reference data would then require coding and matching with the food consumption data before it could be used.

Given the higher human capital requirements of PAPI relative to INDDEX24, the scenario in which we assumed all positions were filled by in-country personnel, while maintaining the small sample sizes of the validation studies, resulted in higher costs of conducting the 24HR using INDDEX24 compared with the PAPI. This finding highlights the importance of considering the context and scale of the dietary recall survey when determining whether using INDDEX24 or a PAPI modality might be the more economical option. When conducting a dietary recall with a very small sample size, as in the validation studies, and where personnel costs are low, the cost savings associated with using the INDDEX24 Platform for some activities may be outweighed by avoiding non-personnel costs like purchasing equipment and a subscription to a mobile platform when using the PAPI modality.

However, our modelling of the cost of national-scale 24HR surveys, which also assumed all positions were filled by in-country personnel, showed that, with scale, the cost per respondent tipped in favour of the INDDEX24 Platform in both Viet Nam and Burkina Faso. This was because some of the fixed or lumpy costs associated with using INDDEX24 (e.g. purchasing equipment and a mobile platform subscription) were spread across many more survey respondents, while the higher personnel requirements, and hence costs, of data entry and data cleaning, processing, and preparation for PAPI were increased in proportion to the scaling of activities. The only other study of which we are aware that estimated the cost of conducting national-scale 24HR surveys in LMIC found that, using the PAPI modality, a single-round 24HR of 8500 households would cost about $178/household in South Asia and or about $247/household in sub-Saharan Africa^(5)^. These estimates are higher than our national-scale PAPI cost estimates of $137/respondent in Viet Nam and $148/respondent in Burkina Faso, which may be partly attributed to the household-level nature of the Fiedler *et al.* estimates and other potential differences in underlying assumptions about study personnel, the duration of the survey, other survey modules, etc.

It is important to interpret the results of these studies in the context of study limitations. The cost studies were not done independently of the validation studies, and it was sometimes difficult to disentangle time spent on work related to the validation study from work that would happen for stand-alone 24HR. As a result, estimates of the person days required to complete certain activities (e.g. management, discussions related to overall study design) may be overestimated. Related, as this was the first full deployment of the INDDEX24 Platform, time was spent working out bugs in the system for both the mobile app and the FMDB. It was sometimes challenging to net out the time spent correcting these bugs during the run-up to the validation study, during data collection and post-data collection, but we can assume future users of the INDDEX24 Platform will not face these additional time costs. Also, the preparation of dietary reference data that occurred prior to the 24HR were then used for both the INDDEX24 and PAPI modalities. For the PAPI modality, while the food list, probes and portion size estimation methods need to be defined in advance, it may be more common for the bulk of dietary reference data work to occur after data collection, which could reduce the cost of developing dietary reference data since the work could focus only on foods, standard recipes and portion sizes that were reported by respondents during data collection. However, doing this work after data collection would also increase the time between the end of data collection and when data are ready to be analysed. Finally, the data for these cost studies were collected from geographically small areas of each country, and the respondents resided in mostly rural settings. While we strove to define national scenarios that were more reflective of the diversity of contexts in which a 24HR might also be conducted, the results of the primary analysis and, to some extent, the assumptions that underpin the national scenarios, reflect the context in which the validation studies took place.

Nevertheless, the cost studies were carried out in two different countries with contextual differences, including variability of diets, population education/literacy rates, wage rates and other factors; this enhances the external validity of our findings. Moreover, the position-specific time and expenditures required to complete each of the carefully defined activities associated with conducting the 24HR were, to the extent possible, recorded in real time. This approach not only allowed for detailed estimates of activity-specific resource requirements that other researchers will be able to use in planning for 24HR but also represents a methodological improvement over previous costing studies of 24HR that have relied on budgets to estimate costs.

These cost studies fill an important gap in knowledge on the cost of conducting 24HR in LMIC and how those costs might vary depending on the modality of data collection. Increasing the availability of high-quality, individual-level dietary recall data in LMIC will require innovative strategies that reduce the barriers associated with collecting, processing and analysing these data. INDDEX24 is a novel dietary assessment platform that provides a coordinated, streamlined approach for electronically collecting data, housing and sharing dietary reference data, and producing automated reports to facilitate data processing and analysis. This cost study showed that, compared with using PAPI, INDDEX24 is a lower cost option for collecting 24HR data in most circumstances. With continued technological improvements to the Platform and as the FMDB becomes a viable source of dietary reference data, the cost of collecting 24HR data using INDDEX24 will further decline. By easing resource requirements, INDDEX24 may facilitate the increased collection and use of individual dietary recall data in LMIC.

## Supplementary material

For supplementary material accompanying this paper visit https://doi.org/10.1017/S0007114522001362. click here to view supplementary material
