# Supplementary material for: The cost and cost efficiency of conducting a 24-h dietary recall using INDDEX24, a mobile dietary assessment platform, compared with pen-and-paper interview in Viet Nam and Burkina Faso
Source: Br J Nutr. 2022 May 5;129(3):535–49. doi: 10.1017/S0007114522001362 (PMC9876804; doi:10.1017/S0007114522001362)
Supplement: Supplementary file 1 [file S0007114522001362sup001.docx]

The cost and cost-efficiency of conducting a 24-hour dietary recall using INDDEX24, a mobile dietary assessment platform, compared to pen-and-paper in Viet Nam and Burkina Faso

**Supplemental Figures**


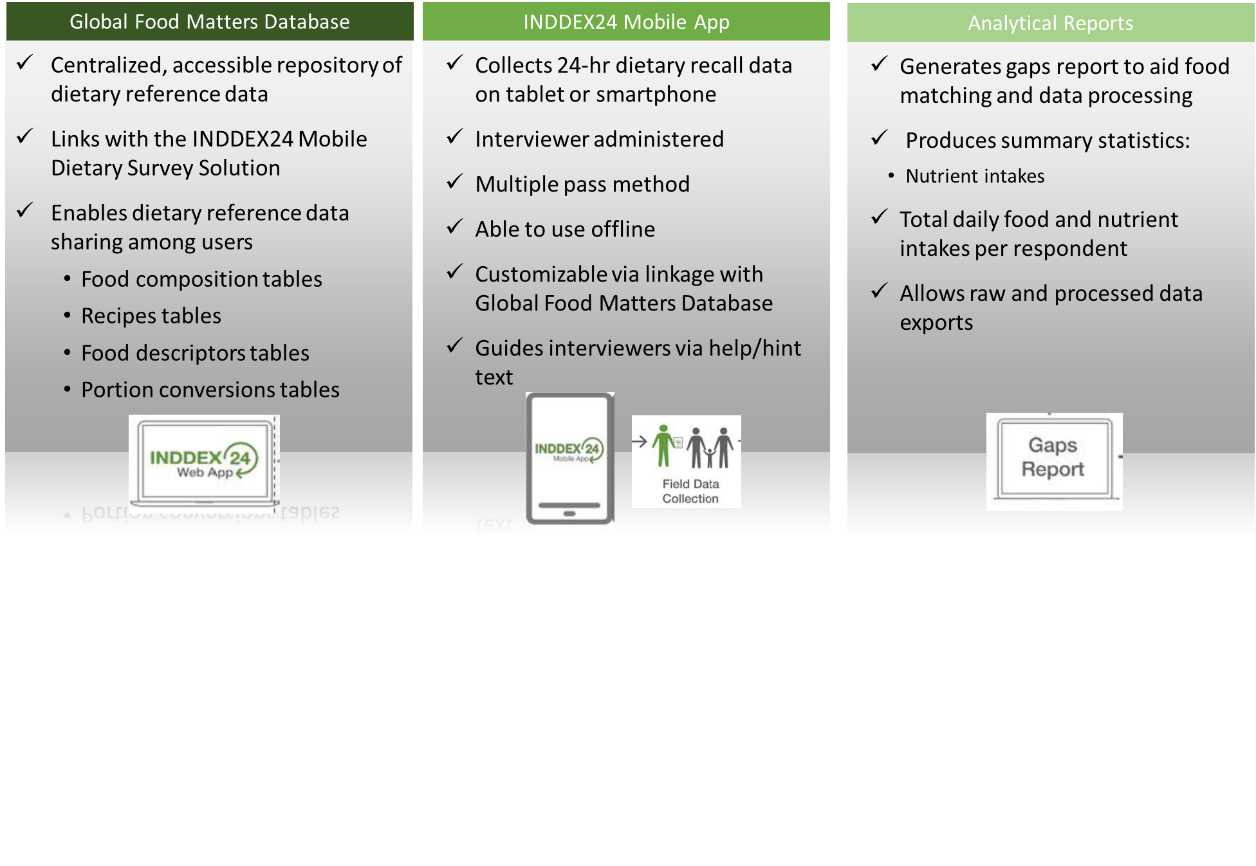


Supplemental Figure S1. Key features of INDDEX24 Dietary Assessment Platform


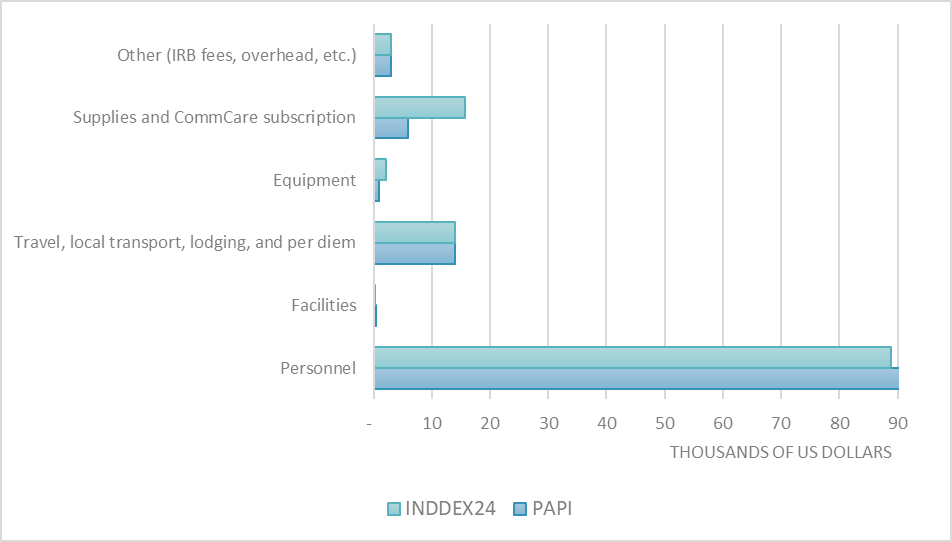


Supplemental Figure S2. Economic cost of conducting a 24hr dietary recall using INDDEX24 and PAPI by cost center: Viet Nam


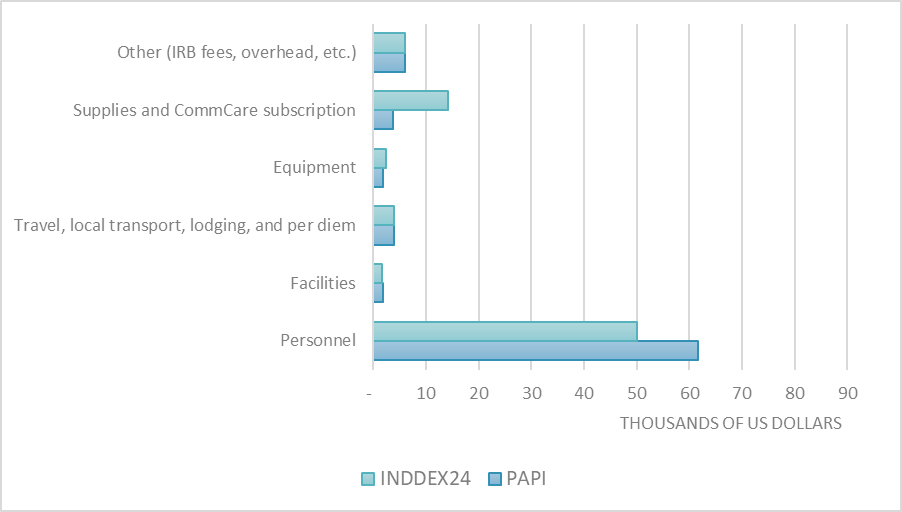


Supplemental Figure S3. Economic cost of conducting a 24hr dietary recall using INDDEX24 and PAPI by cost center: Burkina Faso

**Supplemental Tables**

Supplemental Table S1. INDDEX24 and PAPI 24hr dietary recall survey activities, sub-activities^1^, and sub-activity components

| Primary activity | | Sub-activities | | Components | |
| --- | --- | --- | --- | --- | --- |
| Preparation of dietary data inputs | | Develop food and recipe lists and tags/probes | | 1. Develop, review, and revise draft food and recipe listing based on existing resources, including review of food and recipe listing by national experts to 1) identify any additional gaps or redundancies, 2) rank frequency of consumption of foods and recipes, 3) map all foods and recipes to PSEMs, 4) identify missing conversion factors, 5) fill out other aspects of the food and recipe listing | |
|  | |  | | 1. Identify key tags/descriptors by food group and sub-food group and food, as appropriate | |
|  | |  | | 1. Apply key tags to individual foods and put in appropriate format | |
|  | |  | | 1. Review issues in local language/English and edit tags | |
|  | | Prepare food composition table | | 1. Add energy and nutrient information to the Food Composition tab in the INDDEX24 template based on 1) existing FCT, 2) application of yield, 3) nutrient information borrowed from other sources, and 4) other FCT-related work | |
|  | | Develop standard recipes density factors | | 1. Develop priority standard recipes and density factors based on the frequency of consumption ranking in the food and recipe listing exercise | |
|  | |  | | 1. Format standard recipe information and ingredient proportions | |
|  | |  | | 1. Develop local edible portions for any new foods identified in the food and recipe listing | |
|  | | Identify PSEM/conversion factors | | 1. Make PSEM assignment (includes discussing clustering/allowable substitution of photos and assignment of all foods and recipes to each method, and cut/shape/size of food) | |
|  | |  | | 1. Based on PSEM assignment, develop or identify density factors for all foods that use proxy method | |
|  | |  | | 1. Based on PSEM assignments, take photos of select foods (include buying foods, acquiring photo equipment, setting up space, taking photos) | |
|  | |  | | 1. Develop final conversion calculations for each food item | |
|  | | Compile inputs | | 1. Transfer and finalize formatting of FCT, recipes, tags, and conversion factors to INDDEX24/PAPI template | |
| Survey preparation | | Design paper questionnaires (PAPI only) | | 1. Create and review data collection form for paper 24h dietary recall | |
|  | | Develop data entry form (INDDEX24 only) | | 1. Develop and review data entry form for 24-hour dietary recall | |
|  | | Pilot mobile app/paper questionnaires | | 1. Logistics and prep for field testing of mobile app modules/paper forms | |
|  | |  |  | 1. Pilot mobile app and 24-hr recall module | |
|  | |  | | 1. Incorporate feedback from pilots | |
|  | | Develop manuals for enumerators | | 1. Adapt generic guidance manual to survey context for enumerators | |
|  | |  | | 1. Develop training materials (e.g., slide deck) | |
|  | | Translate forms and manuals | | 1. Translate 24hr dietary recall form, consent form, recruitment form, and training materials | |
|  | | Print survey instruments/questionnaires | | 1. Print questionnaires, consent forms, manuals, training material, etc. | |
|  | | Print photo atlas | | 1. Print photo atlas | |
|  | | Receive ethical approval | | 1. Prepare and submit for ethical approval | |
|  | | Purchase and prepare supplies and equipment | | 1. Tablets | |
|  | |  |  | 1. Cases | |
|  | |  |  | 1. Data | |
|  | |  | | 1. SIM cards | |
|  | |  | | 1. Pens and notebooks | |
|  | |  | | 1. Plastic folders | |
|  | |  | | 1. Backpacks and bags for scales | |
|  | |  | | 1. Kitchen scales | |
|  | |  | | 1. Standard weight | |
|  | |  | | 1. Play dough | |
|  | |  | | 1. Proxy for food | |
|  | |  | | 1. Plastic container | |
|  | |  | | 1. Other household measures | |
|  | |  | | 1. Portable hard drive | |
|  | |  | | 1. Set up tablets (create user accounts, load apps, etc.) | |
|  | | Purchase CommCare subscription (INDDEX24 only) | | 1. Purchase 12-month CommCare subscription | |
| Training | | Supervisor training | | 1. Supervisor training on 24-hour recall method | |
|  | | Enumerator training | | 1. Enumerator training on 24-hour recall method and use of tablets/paper forms | |
|  | |  | | 1. Enumerator refresher training | |
|  | | Data entry clerk training | | 1. Data entry clerk training | |
| Survey execution | | Household listing and sampling of eligible participants | | 1. Develop full community sampling frame | |
|  | |  | | 1. Recruit and select respondents | |
|  | | Incentives | | 1. Incentives for participants | |
|  | |  | | 1. Incentives for local collaborators | |
|  | | Data collection and field supervision | | 1. Collect data | |
|  | |  |  | 1. Supervise data collection | |
|  | | Electronic data monitoring (INDDEX24 only) | | 1. Monitor electronic data | |
| Data entry | | Data entry and supervision (PAPI only) | | 1. Enter data and supervise data entry | |
| Data cleaning, processing, and preparation for analysis | | Data cleaning, processing (food matching, gap filling, etc.), and preparation for analysis | | 1. Clean, process (food matching, gap filling, etc.), and prepare data for analysis | |
| Administration | | Management and oversight | | 1. Overall survey management and oversight | |
|  | |  | | 1. On the ground survey management and implementation | |
|  | | International travel to the field | | 1. Travel to the field | |
|  | | Lodging/per diem for international personnel | | 1. Lodging and per diem | |
|  | | Overhead | | 1. Overhead | |

24HR, 24hr dietary recall; FCT, food composition table; PAPI, pen and paper personal interview; PSEM, portion size estimation method.

^1^Sub-activities apply to both INDDEX24 and PAPI unless otherwise stated.

Supplemental Table S2. National scenario cost modeling assumptions

| **Activity** | **Sub-activity** | **Viet Nam** | **Burkina Faso** |
| --- | --- | --- | --- |
| Prepare dietary data inputs | All | Validation study dietary data input preparation work was nationally representative; no difference in cost. | Validation study dietary data inputs would cover ~60% of inputs needed at the national level; cost of preparing dietary data inputs estimated to increase by 40%. |
| Survey preparation | Design paper questionnaire for 24hr dietary recall | No difference in cost. | No difference in cost. |
|  | Develop data entry form for 24hr dietary recall | No difference in cost. | No difference in cost. |
|  | Pilot mobile app/paper questionnaires and pilot data collection process | Assumed to happen in each subnational "hub"; piloting costs multiplied by 6 plus 1 extra day for enumerator piloting | Assumed to happen in each subnational "hub"; piloting costs multiplied by 5 plus 1 extra day for enumerator piloting |
|  | Develop manuals and training materials | Assume generic manuals and training materials available via INDDEX24; cost of adaptation 1 day | No difference in cost. |
|  | Translate 24hr dietary recall form, consent form, recruitment form, and training materials | Translation into Vietnamese sufficient for entire country; no difference in cost. | Translated into 3 additional languages; translation costs multiplied by 3. |
|  | Print survey instruments | Linear scale up based on number of participants. | Linear scale up based on number of participants. |
|  | Print photo atlas | Linear scale up based on number of enumerators. | Base cost increase by 40% to reflect expanded dietary data inputs then linear scale up based on number of enumerators. |
|  | Receive ethical approval | No difference in cost. | National surveys require a statistical visa. The visa itself is free, but additional cost of 300,000 CFA to organize the meeting of the statistical visa committee. |
|  | Purchase and prepare supplies and equipment | Linear scale up based on number of enumerators. | Linear scale up based on number of enumerators. |
|  | Purchase CommCare subscription | Reduce to $5,000 to reflect expectations about future INDDEX24 user fees. | Reduce to $5,000 to reflect expectations about future INDDEX24 user fees. |
| Training | Field coordinator training | Centralized; 4 days | Centralized; 4 days |
|  | Field supervisor training | Centralized; 10 days | Centralized; 10 days |
|  | Enumerator training | Centralized; 12 days | Centralized; 12 days |
|  | Enumerator refresher training | At regional hubs; 1 day | At regional hubs; 1 day |
|  | Data entry clerk training | Centralized; 2 days | Centralized; 2 days |
| Survey execution | Household listing and sampling of eligible participants | Linear scale up based on number of participants. | Linear scale up based on number of participants. |
|  | Incentives | Zero | Zero |
|  | Recruitment, data collection, and field supervision | Linear scale up (excluding staff per diems) based on number of enumerators and supervisors. Local transport scaled up based on number of respondents. | Linear scale up (excluding staff per diems) based on number of enumerators and supervisors. Local transport scaled up based on number of respondents. |
|  | Electronic data monitoring | Linear scale up based on number of participants. | Linear scale up based on number of participants. |
| Data entry | Data entry and supervision | Linear scale up based on number of data entry clerks/supervisors. | Linear scale up based on number of data entry clerks/supervisors. |
| Data cleaning, processing, and preparation for analysis | Data cleaning, processing (food matching, gap filling, etc.), and preparation for analysis | Data cleaning - linear scale up based on number of participants; data processing and preparation - no difference in cost. | Data cleaning - linear scale up based on number of participants; data processing - 40% increase to match increase in dietary data inputs; data preparation - no difference in cost. |
| Administration | Management and oversight | Project lead: 30% FTE during pre-data collection and data collection (7 months); 30% FTE for 4 months post-data collection. Deputy at 100% FTE for pre-data collection, data collection, and post data collection. | Project lead: 30% FTE during pre-data collection and data collection (7 months); 30% FTE for 4 months post-data collection. Deputy at 100% FTE for pre-data collection, data collection, and post data collection. |
|  | International travel to the field | zero (all in-country staff) | zero (all in-country staff) |
|  | Lodging and per diem for international personnel | zero (all in-country staff) | zero (all in-country staff) |
|  | Overhead/Unforeseen expenses | 10% of total budget | 10% of total budget |

Supplemental Table S3.Time (human capital) and non-time (non-human capital) costs of conducting a 24hr dietary recall using INDDEX24 and PAPI: Viet Nam

|  | |  | | INDDEX24 | | | | | | PAPI | | | | |
| --- | --- | --- | --- | --- | --- | --- | --- | --- | --- | --- | --- | --- | --- | --- |
|  | |  | | Time | | Time cost | Non-time cost | | | Time | | Time cost | | Non-time cost |
| Primary activity | | Sub-activities | | (person days) | | (2019 USD) | (2019 USD) | | | (person days) | | (2019 USD) | | (2019 USD) |
| Preparation of dietary data inputs | Develop food and recipe lists and tags/probes | | 137.0 | | 14,098 | | | 0 | 137.0 | | 14,098 | | 0 | |
|  | Prepare food composition table | | 88.2 | | 1,005 | | | 0 | 88.2 | | 1,005 | | 0 | |
|  | Develop standard recipes density factors | | 341.0 | | 8,401 | | | 1,937 | 341.0 | | 8,401 | | 1,937 | |
|  | Identify PSEM/conversion factors | | 253.0 | | 12,366 | | | 86 | 253.0 | | 12,366 | | 86 | |
|  | Compile and format dietary reference data | | 37.0 | | 3,659 | | | 0 | 37.0 | | 3,659 | | 0 | |
|  | Sub-total | | 856.2 | | 39,529 | | | 2,023 | 856.2 | | 39,529 | | 2,023 | |
| Survey preparation | Design paper questionnaire for 24HR | | 0.0 | | 0 | | | 0 | 6.0 | | 2,157 | | 0 | |
|  | Develop data entry form for 24HR | | 0.0 | | 0 | | | 0 | 14.6 | | 5,423 | | 0 | |
|  | Pilot mobile app/paper questionnaires | | 6.1 | | 2,121 | | | 0 | 4.6 | | 1,118 | | 0 | |
|  | Develop manuals and training materials | | 6.1 | | 2,285 | | | 0 | 5.1 | | 1,912 | | 0 | |
|  | Translate forms and training materials | | 20.7 | | 1,325 | | | 0 | 20.7 | | 1,325 | | 0 | |
|  | Print survey instruments/questionnaires | | 0.3 | | 18 | | | 301 | 1.0 | | 70 | | 646 | |
|  | Print photo atlas | | 1.0 | | 70 | | | 424 | 1.0 | | 70 | | 424 | |
|  | Receive ethical approval | | 3.5 | | 732 | | | 858 | 3.5 | | 732 | | 858 | |
|  | Purchase and prepare supplies and equipment | | 2.6 | | 319 | | | 1,715 | 0.5 | | 35 | | 788 | |
|  | Purchase CommCare subscription | | 0.0 | | 0 | | | 10,000 | 0.0 | | 0 | | 0 | |
|  | Sub-total | | 40.3 | | 6,870 | | | 13,299 | 57.1 | | 12,842 | | 2,716 | |
| Training | Supervisor training | | 0.0 | | 0 | | | 0 | 0.0 | | 0 | | 0 | |
|  | Enumerator training | | 58.0 | | 4,840 | | | 428 | 40.0 | | 3,352 | | 428 | |
|  | Data entry clerk training | | 0.0 | | 0 | | | 0 | 12.0 | | 979 | | 0 | |
|  | Sub-total | | 58.0 | | 4,840 | | | 428 | 52.0 | | 4,330 | | 428 | |
| Survey execution | Household listing and sampling of eligible participants | | 83.6 | | 1,770 | | | 872 | 83.6 | | 1,770 | | 872 | |
|  | Incentives | | 0.0 | | 0 | | | 1,782 | 0.0 | | 0 | | 1,782 | |
|  | Data collection and field supervision | | 99.9 | | 3,409 | | | 4,918 | 101.5 | | 3,420 | | 4,918 | |
|  | Electronic data monitoring | | 5.6 | | 2,089 | | | 0 | 0.0 | | 0 | | 0 | |
|  | Sub-total | | 189.1 | | 7,269 | | | 7,571 | 185.1 | | 5,191 | | 7,571 | |
| Data entry | Data entry and supervision | | 0.0 | | 0 | | | 0 | 22.6 | | 570 | | 835 | |
|  | Sub-total | | 0.0 | | 0 | | | 0 | 22.6 | | 570 | | 835 | |
| Data cleaning, processing, and preparation | Data cleaning, processing (food matching, gap filling, etc.), and preparation for analysis | | 46.9 | | 8,500 | | | 0 | 73.3 | | 19,440 | | 0 | |
|  | Sub-total | | 46.9 | | 8,500 | | | 0 | 73.3 | | 19,440 | | 0 | |
| Administration | Management and oversight | | 65.8 | | 14,795 | | | 0 | 73.3 | | 19,127 | | 0 | |
|  | International travel to the field | | 0.0 | | 0 | | | 1,700 | 0.0 | | 0 | | 1,700 | |
|  | Lodging/per diem for international personnel | | 0.0 | | 0 | | | 2,027 | 0.0 | | 0 | | 2,027 | |
|  | Overhead | | 0.0 | | 0 | | | 2,156 | 0.0 | | 0 | | 2,156 | |
|  | Sub-total | | 65.8 | | 14,795 | | | 5,882 | 73.3 | | 19,127 | | 5,882 | |
| Totals | Prepare dietary reference data | | 856.2 | | 39,529 | | | 2,023 | 856.2 | | 39,529 | | 2,023 | |
|  | Survey preparation | | 40.3 | | 6,870 | | | 13,299 | 57.1 | | 12,842 | | 2,716 | |
|  | Training | | 58.0 | | 4,840 | | | 428 | 52.0 | | 4,330 | | 428 | |
|  | Survey execution | | 189.1 | | 7,269 | | | 7,571 | 185.1 | | 5,191 | | 7,571 | |
|  | Data entry | | 0.0 | | 0 | | | 0 | 22.6 | | 570 | | 835 | |
|  | Data cleaning, processing, & preparation | | 46.9 | | 8,500 | | | 0 | 73.3 | | 19,440 | | 0 | |
|  | Administration | | 65.8 | | 14,795 | | | 5,882 | 73.3 | | 19,127 | | 5,882 | |
|  | Grand total | | 1256.2 | | 81,801 | | | 29,203 | 1319.5 | | 101,028 | | 19,455 | |
|  | Number of respondents | | 147 | | 147 | | | 147 | 147 | | 147 | | 147 | |
|  | Total per respondent | | 8.5 | | 556 | | | 199 | 9.0 | | 687 | | 132 | |

24HR, 24hr dietary recall; INDDEX24, INDDEX24 Dietary Assessment Platform; PAPI, pen-and-paper interview; PSEM, portion size estimation method; USD, US dollars.

Supplemental Table S4. Time (human capital) and non-time (non-human capital) costs of conducting a 24hr dietary recall using INDDEX24 and PAPI: Burkina Faso

|  | |  | | INDDEX24 | | | | | | PAPI | | | | |
| --- | --- | --- | --- | --- | --- | --- | --- | --- | --- | --- | --- | --- | --- | --- |
|  | |  | | Time | | Time cost | Non-time cost | | | Time | | Time cost | | Non-time cost |
| Primary activity | | Sub-activities | | (person days) | | (2019 USD) | (2019 USD) | | | (person days) | | (2019 USD) | | (2019 USD) |
| Preparation of dietary data inputs | Develop food and recipe lists and tags/probes | | 21.0 | | 2,112 | | | 0 | 21.0 | | 2,112 | | 0 | |
|  | Prepare food composition table | | 0.0 | | 0 | | | 0 | 0.0 | | 0 | | 0 | |
|  | Develop standard recipes density factors | | 69.9 | | 5,853 | | | 1,660 | 69.9 | | 5,853 | | 1,660 | |
|  | Identify PSEM/conversion factors | | 2.9 | | 503 | | | 1,563 | 2.9 | | 503 | | 1,563 | |
|  | Compile and format dietary reference data | | 9.4 | | 1,587 | | | 0 | 9.4 | | 1,587 | | 0 | |
|  | Sub-total | | 103.1 | | 10,055 | | | 3,223 | 103.1 | | 10,055 | | 3,223 | |
| Survey preparation | Design paper questionnaire for 24HR | | 0 | | 0 | | | 0 | 5.5 | | 963 | | 0 | |
|  | Develop data entry form for 24HR | | 0 | | 0 | | | 0 | 14.0 | | 4,525 | | 0 | |
|  | Pilot mobile app/paper questionnaires | | 5.2 | | 588 | | | 0 | 5.1 | | 567 | | 0 | |
|  | Develop manuals and training materials | | 5.1 | | 893 | | | 0 | 5.1 | | 893 | | 0 | |
|  | Translate forms and training materials | | 21.4 | | 1,973 | | | 0 | 21.3 | | 1,924 | | 0 | |
|  | Print survey instruments/questionnaires | | 0.4 | | 16 | | | 20 | 0.8 | | 93 | | 132 | |
|  | Print photo atlas | | 0.3 | | 44 | | | 319 | 0.3 | | 44 | | 319 | |
|  | Receive ethical approval | | 3.5 | | 732 | | | 1,330 | 3.5 | | 732 | | 1,330 | |
|  | Purchase and prepare supplies and equipment | | 2.3 | | 474 | | | 1,808 | 0.9 | | 133 | | 760 | |
|  | Purchase CommCare subscription | | 0.0 | | 0 | | | 10,000 | 0.0 | | 0 | | 0 | |
|  | Sub-total | | 38.1 | | 4,719 | | | 13,478 | 56.4 | | 9,872 | | 2,541 | |
| Training | Supervisor training | | 29.0 | | 2,473 | | | 596 | 29.0 | | 2,473 | | 596 | |
|  | Enumerator training | | 52.0 | | 3,343 | | | 954 | 50.4 | | 3,272 | | 954 | |
|  | Data entry clerk training | | 0.0 | | 0 | | | 0 | 6.5 | | 284 | | 85 | |
|  | Sub-total | | 81.0 | | 5,816 | | | 1,550 | 85.9 | | 6,028 | | 1,635 | |
| Survey execution | Household listing and sampling of eligible participants | | 54.8 | | 2,737 | | | 890 | 54.8 | | 2,737 | | 890 | |
|  | Incentives | | 0.0 | | 0 | | | 1,124 | 0.0 | | 0 | | 1,124 | |
|  | Data collection and field supervision | | 116.7 | | 5,290 | | | 3,140 | 117.7 | | 5,293 | | 3,140 | |
|  | Electronic data monitoring | | 5.4 | | 1,943 | | | 0 | 0.3 | | 55 | | 0 | |
|  | Sub-total | | 176.9 | | 9,971 | | | 5,154 | 172.8 | | 8,085 | | 5,154 | |
| Data entry | Data entry and supervision | | 0.0 | | 0 | | | 0 | 24.5 | | 594 | | 174 | |
|  | Sub-total | | 0.0 | | 0 | | | 0 | 24.5 | | 594 | | 174 | |
| Data cleaning, processing, and preparation | Data cleaning, processing (food matching, gap filling, etc.), and preparation for analysis | | 28.8 | | 8,370 | | | 0 | 40.4 | | 12,001 | | 0 | |
|  | Sub-total | | 28.8 | | 8,370 | | | 0 | 40.4 | | 12,001 | | 0 | |
| Administration | Management and oversight | | 35.3 | | 11,194 | | | 0 | 42.8 | | 15,526 | | 0 | |
|  | International travel to the field | | 0.0 | | 0 | | | 0 | 0.0 | | 0 | | 0 | |
|  | Lodging/per diem for international personnel | | 0.0 | | 0 | | | 0 | 0.0 | | 0 | | 0 | |
|  | Overhead | | 0.0 | | 0 | | | 4,576 | 0.0 | | 0 | | 4,576 | |
|  | Sub-total | | 35.3 | | 11,194 | | | 4,576 | 42.8 | | 15,526 | | 4,576 | |
| Totals | Prepare dietary reference data | | 103.1 | | 10,055 | | | 3,223 | 103.1 | | 10,055 | | 3,223 | |
|  | Survey preparation | | 38.1 | | 4,719 | | | 13,478 | 56.4 | | 9,872 | | 2,541 | |
|  | Training | | 81.0 | | 5,816 | | | 1,550 | 85.9 | | 6,028 | | 1,635 | |
|  | Survey execution | | 176.9 | | 9,971 | | | 5,154 | 172.8 | | 8,085 | | 5,154 | |
|  | Data entry | | 0.0 | | 0 | | | 0 | 24.5 | | 594 | | 174 | |
|  | Data cleaning, processing, and preparation | | 28.8 | | 8,370 | | | 0 | 40.4 | | 12,001 | | 0 | |
|  | Administration | | 35.3 | | 11,194 | | | 4,576 | 42.8 | | 15,526 | | 4,576 | |
|  | Grand total | | 463.2 | | 50,124 | | | 27,981 | 526.0 | | 62,162 | | 17,303 | |
|  | Number of respondents | | 145 | | 145 | | | 145 | 146 | | 146 | | 146 | |
|  | Total per respondent | | 3 | | 346 | | | 193 | 4 | | 426 | | 119 | |

24HR, 24hr dietary recall; INDDEX24, INDDEX24 Dietary Assessment Platform; PAPI, pen-and-paper interview; PSEM, portion size estimation method; USD, US dollars.

Supplemental Table S5. Economic cost and cost-efficiency of conducting a 24hr dietary recall using INDDEX24 and PAPI assuming all in-country personnel: Viet Nam

|  |  | INDDEX24 | | PAPI | | Difference^1^ |
| --- | --- | --- | --- | --- | --- | --- |
|  |  | Total cost | Percent of activity total | Total cost | Percent of activity total | INDDEX24-PAPI |
| Primary activity | Sub-activities | (2019 USD) |  | (2019 USD) |  | (2019 USD) |
| Preparation of dietary reference data | Develop food and recipe lists and tags/probes | 7,400 | 26.5% | 7,400 | 26.5% | 0 |
|  | Prepare food composition table | 1,005 | 3.6% | 1,005 | 3.6% | 0 |
|  | Develop standard recipes density factors | 10,338 | 37.1% | 10,338 | 37.1% | 0 |
|  | Identify PSEM/conversion factors | 7,299 | 26.2% | 7,299 | 26.2% | 0 |
|  | Compile and format dietary reference data | 1,840 | 6.6% | 1,840 | 6.6% | 0 |
|  | Sub-total | 27,882 |  | 27,882 |  | 0 |
| Survey preparation | Design paper questionnaire for 24HR | 0 | 0.0% | 400 | 6.6% | -400 |
|  | Develop data entry form for 24HR | 0 | 0.0% | 518 | 8.6% | -518 |
|  | Pilot mobile app/paper questionnaires | 454 | 2.9% | 436 | 7.2% | 18 |
|  | Develop manuals and training materials | 429 | 2.7% | 359 | 5.9% | 70 |
|  | Translate forms and training materials | 1,325 | 8.4% | 1,325 | 22.0% | 0 |
|  | Print survey instruments/questionnaires | 319 | 2.0% | 716 | 11.9% | -397 |
|  | Print photo atlas | 494 | 3.1% | 494 | 8.2% | 0 |
|  | Receive ethical approval | 963 | 6.1% | 963 | 16.0% | 0 |
|  | Purchase and prepare supplies and equipment | 1,858 | 11.7% | 823 | 13.6% | 1,035 |
|  | Purchase CommCare subscription | 10,000 | 63.1% | 0 | 0.0% | 10,000 |
|  | Sub-total | 15,841 |  | 6,034 |  | 9,807 |
| Training | Supervisor training^2^ | 0 | 0.0% | 0 | 0.0% | 0 |
|  | Enumerator training | 3,297 | 100.0% | 2,415 | 86.6% | 882 |
|  | Data entry clerk training | 0 | 0.0% | 372 | 13.4% | -372 |
|  | Sub-total | 3,297 |  | 2,788 |  | 509 |
| Survey execution | Household listing and sampling of eligible participants | 2,642 | 20.1% | 2,642 | 20.7% | 0 |
|  | Incentives | 1,782 | 13.6% | 1,782 | 14.0% | 0 |
|  | Data collection and field supervision | 8,327 | 63.4% | 8,338 | 65.3% | -11 |
|  | Electronic data monitoring | 392 | 3.0% | 0 | 0.0% | 392 |
|  | Sub-total | 13,143 |  | 12,762 |  | 381 |
| Data entry | Data entry and supervision | 0 | 0.0% | 1,405 | 100.0% | -1,405 |
|  | Sub-total | 0 |  | 1,405 |  | -1,405 |
| Data cleaning, processing, and preparation | Data cleaning, processing (food matching, gap filling, etc.), and preparation for analysis | 2,187 | 100.0% | 3,327 | 100.0% | -1,140 |
|  | Sub-total | 2,187 |  | 3,327 |  | -1,140 |
| Administration | Management and oversight | 4,979 | 45.8% | 5,732 | 49.4% | -753 |
|  | International travel to the field | 1,700 | 15.7% | 1,700 | 14.6% | 0 |
|  | Lodging/per diem for international personnel | 2,027 | 18.7% | 2,027 | 17.4% | 0 |
|  | Overhead | 2,156 | 19.8% | 2,156 | 18.6% | 0 |
|  | Sub-total | 10,861 |  | 11,614 |  | -753 |
| Totals | Prepare dietary reference data | 27,882 | 38.1% | 27,882 | 42.4% | 0 |
|  | Survey preparation | 15,841 | 21.6% | 6,034 | 9.2% | 9,807 |
|  | Training | 3,297 | 4.5% | 2,788 | 4.2% | 509 |
|  | Survey execution | 13,143 | 18.0% | 12,762 | 19.4% | 381 |
|  | Data entry | 0 | 0.0% | 1,405 | 2.1% | -1,405 |
|  | Data cleaning, processing, and preparation | 2,187 | 3.0% | 3,327 | 5.1% | -1,140 |
|  | Administration | 10,861 | 14.8% | 11,614 | 17.6% | -753 |
|  | Grand total | 73,211 | 100.0% | 65,812 | 100.0% | 7,399 |
|  | Number of respondents | 147 |  | 147 |  | 0 |
|  | Total per respondent | 498 |  | 448 |  | 50 |

24HR, 24hr dietary recall; INDDEX24, INDDEX24 Dietary Assessment Platform; PAPI, pen-and-paper interview; PSEM, portion size estimation method; USD, US dollars.

^1^The difference is calculated as the cost of INDDEX24 minus the cost of PAPI.

^2^Due to time constraints, supervisor training did not take place as a separate activity in Viet Nam.

Supplemental Table S6. Economic cost and cost-efficiency of conducting a 24hr dietary recall using INDDEX24 and PAPI assuming all in-country personnel: Burkina Faso

|  |  | INDDEX24 | | PAPI | | Difference^1^ |
| --- | --- | --- | --- | --- | --- | --- |
|  |  | Total cost | Percent of activity total | Total cost | Percent of activity total | INDDEX24-PAPI |
| Primary activity | Sub-activities | (2019 USD) |  | (2019 USD) |  | (2019 USD) |
| Preparation of dietary reference data | Develop food and recipe lists and tags/probes | 2,112 | 16.4% | 2,112 | 16.4% | 0 |
|  | Prepare food composition table | 0 | 0.0% | 0 | 0.0% | 0 |
|  | Develop standard recipes density factors | 7,513 | 58.5% | 7,513 | 58.5% | 0 |
|  | Identify PSEM/conversion factors | 2,066 | 16.1% | 2,066 | 16.1% | 0 |
|  | Compile and format dietary reference data | 1,156 | 9.0% | 1,156 | 9.0% | 0 |
|  | Sub-total | 12,847 |  | 12,847 |  | 0 |
| Survey preparation | Design paper questionnaire for 24HR | 0 | 0.0% | 963 | 12.1% | -963 |
|  | Develop data entry form for 24HR | 0 | 0.0% | 657 | 8.3% | -657 |
|  | Pilot mobile app/paper questionnaires | 588 | 3.4% | 567 | 7.2% | 21 |
|  | Develop manuals and training materials | 893 | 5.2% | 893 | 11.3% | 0 |
|  | Translate forms and training materials | 1,942 | 11.2% | 1,924 | 24.3% | 18 |
|  | Print survey instruments/questionnaires | 36 | 0.2% | 224 | 2.8% | -188 |
|  | Print photo atlas | 363 | 2.1% | 363 | 4.6% | 0 |
|  | Receive ethical approval | 1,500 | 8.7% | 1,500 | 18.9% | 0 |
|  | Purchase and prepare supplies and equipment | 1,991 | 11.5% | 833 | 10.5% | 1,159 |
|  | Purchase CommCare subscription | 10,000 | 57.8% | 0 | 0.0% | 10,000 |
|  | Sub-total | 17,313 |  | 7,923 |  | 9,390 |
| Training | Supervisor training | 3,069 | 41.7% | 3,069 | 40.0% | 0 |
|  | Enumerator training | 4,297 | 58.3% | 4,226 | 55.1% | 71 |
|  | Data entry clerk training | 0 | 0.0% | 369 | 4.8% | -369 |
|  | Sub-total | 7,366 |  | 7,664 |  | -298 |
| Survey execution | Household listing and sampling of eligible participants | 3,626 | 26.1% | 3,626 | 27.4% | 0 |
|  | Incentives | 1,124 | 8.1% | 1,124 | 8.5% | 0 |
|  | Data collection and field supervision | 8,431 | 60.6% | 8,434 | 63.7% | -3 |
|  | Electronic data monitoring | 735 | 5.3% | 55 | 0.4% | 680 |
|  | Sub-total | 13,916 |  | 13,238 |  | 678 |
| Data entry | Data entry and supervision | 0 | 0.0% | 768 | 100.0% | -768 |
|  | Sub-total | 0 |  | 768 |  | -768 |
| Data cleaning, processing, and preparation | Data cleaning, processing (food matching, gap filling, etc.), and preparation for analysis | 3,437 | 100.0% | 4,690 | 100.0% | -1,253 |
|  | Sub-total | 3,437 |  | 4,690 |  | -1,253 |
| Administration | Management and oversight | 6,608 | 59.1% | 8,189 | 64.2% | -1,581 |
|  | International travel to the field | 0 | 0.0% | 0 | 0.0% | 0 |
|  | Lodging/per diem for international personnel | 0 | 0.0% | 0 | 0.0% | 0 |
|  | Overhead | 4,576 | 40.9% | 4,576 | 35.8% | 0 |
|  | Sub-total | 11,184 |  | 12,766 |  | -1,581 |
| Totals | Prepare dietary reference data | 12,847 | 19.4% | 12,847 | 21.4% | 0 |
|  | Survey preparation | 17,313 | 26.2% | 7,923 | 13.2% | 9,390 |
|  | Training | 7,366 | 11.1% | 7,664 | 12.8% | -298 |
|  | Survey execution | 13,916 | 21.1% | 13,238 | 22.1% | 678 |
|  | Data entry | 0 | 0.0% | 768 | 1.3% | -768 |
|  | Data cleaning, processing, and preparation | 3,437 | 5.2% | 4,690 | 7.8% | -1,253 |
|  | Administration | 11,184 | 16.9% | 12,766 | 21.3% | -1,581 |
|  | Grand total | 66,063 | 100.0% | 59,895 | 100.0% | 6,168 |
|  | Number of respondents | 145 |  | 146 |  | -1 |
|  | Total per respondent | 456 |  | 410 |  | 45 |

24HR, 24hr dietary recall; INDDEX24, INDDEX24 Dietary Assessment Platform; PAPI, pen-and-paper interview; PSEM, portion size estimation method; USD, US dollars.

^1^The difference is calculated as the cost of INDDEX24 minus the cost of PAPI.

Supplemental Table S7. Time (human capital) and non-time (non-human capital) costs of conducting a national scale 24hr dietary recall using INDDEX24 and PAPI: Viet Nam

|  | |  | | INDDEX24 | | | | | | PAPI | | | | |
| --- | --- | --- | --- | --- | --- | --- | --- | --- | --- | --- | --- | --- | --- | --- |
|  | |  | | Time | | Time cost | Non-time cost | | | Time | | Time cost | | Non-time cost |
| Primary activity | | Sub-activities | | (person days) | | (2019 USD) | (2019 USD) | | | (person days) | | (2019 USD) | | (2019 USD) |
| Preparation of dietary data inputs | Develop food and recipe lists and tags/probes | | 137.0 | | 7,400 | | | 0 | 137.0 | | 7,400 | | 0 | |
|  | Prepare food composition table | | 88.2 | | 1,005 | | | 0 | 88.2 | | 1,005 | | 0 | |
|  | Develop standard recipes density factors | | 341.0 | | 8,401 | | | 1,937 | 341.0 | | 8,401 | | 1,937 | |
|  | Identify PSEM/conversion factors | | 253.0 | | 7,213 | | | 86 | 253.0 | | 7,213 | | 86 | |
|  | Compile and format dietary reference data | | 37.0 | | 1,840 | | | 0 | 37.0 | | 1,840 | | 0 | |
|  | Sub-total | | 856.2 | | 25,859 | | | 2,023 | 856.2 | | 25,859 | | 2,023 | |
| Survey preparation | Design paper questionnaire for 24HR | | 0.0 | | 0 | | | 0 | 6.0 | | 400 | | 0 | |
|  | Develop data entry form for 24HR | | 0.0 | | 0 | | | 0 | 14.6 | | 518 | | 0 | |
|  | Pilot mobile app/paper questionnaires | | 78.8 | | 4,169 | | | 0 | 69.8 | | 4,064 | | 0 | |
|  | Develop manuals and training materials | | 1.0 | | 70 | | | 0 | 5.1 | | 359 | | 0 | |
|  | Translate forms and training materials | | 20.7 | | 1,325 | | | 0 | 20.7 | | 1,325 | | 0 | |
|  | Print survey instruments/questionnaires | | 8.9 | | 625 | | | 10,763 | 35.7 | | 2,500 | | 23,064 | |
|  | Print photo atlas | | 10.5 | | 735 | | | 4,456 | 10.5 | | 735 | | 4,456 | |
|  | Receive ethical approval | | 3.5 | | 105 | | | 858 | 3.5 | | 105 | | 858 | |
|  | Purchase and prepare supplies and equipment | | 35.8 | | 1,994 | | | 24,011 | 7.0 | | 490 | | 11,026 | |
|  | Purchase CommCare subscription | | 0.0 | | 0 | | | 5,000 | 0.0 | | 0 | | 0 | |
|  | Sub-total | | 159.1 | | 9,023 | | | 45,088 | 172.9 | | 10,496 | | 39,404 | |
| Training | Supervisor training | | 302.0 | | 20,195 | | | 1,697 | 302.0 | | 20,195 | | 1,697 | |
|  | Enumerator training | | 884.0 | | 35,498 | | | 8,841 | 884.0 | | 35,498 | | 8,841 | |
|  | Data entry clerk training | | 0.0 | | 0 | | | 0 | 152.0 | | 3,489 | | 799 | |
|  | Sub-total | | 1186.0 | | 55,692 | | | 10,539 | 1338.0 | | 59,181 | | 11,338 | |
| Survey execution | Household listing and sampling of eligible participants | | 2488.5 | | 52,704 | | | 34,917 | 2488.5 | | 52,704 | | 34,917 | |
|  | Incentives | | 0.0 | | 0 | | | 0 | 0.0 | | 0 | | 0 | |
|  | Data collection and field supervision | | 1986.6 | | 68,873 | | | 60,350 | 2043.5 | | 69,282 | | 60,350 | |
|  | Electronic data monitoring | | 200.0 | | 14,003 | | | 0 | 0.0 | | 0 | | 0 | |
|  | Sub-total | | 4675.2 | | 135,579 | | | 95,267 | 4532.0 | | 121,986 | | 95,267 | |
| Data entry | Data entry and supervision | | 0.0 | | 0 | | | 0 | 1950.0 | | 43,535 | | 9,384 | |
|  | Sub-total | | 0.0 | | 0 | | | 0 | 1950.0 | | 43,535 | | 9,384 | |
| Data cleaning, processing, and preparation | Data cleaning, processing (food matching, gap filling, etc.), and preparation for analysis | | 535.5 | | 24,972 | | | 0 | 2159.5 | | 98,057 | | 0 | |
|  | Sub-total | | 535.5 | | 24,972 | | | 0 | 2159.5 | | 98,057 | | 0 | |
| Administration | Management and oversight | | 353.5 | | 26,824 | | | 0 | 353.5 | | 26,824 | | 0 | |
|  | International travel to the field | | 0.0 | | 0 | | | 0 | 0.0 | | 0 | | 0 | |
|  | Lodging/per diem for international personnel | | 0.0 | | 0 | | | 0 | 0.0 | | 0 | | 0 | |
|  | Overhead | | 0.0 | | 0 | | | 46,401 | 0.0 | | 0 | | 57,650 | |
|  | Sub-total | | 353.5 | | 26,824 | | | 46,401 | 353.5 | | 26,824 | | 57,650 | |
| Totals | Prepare dietary reference data | | 856.2 | | 25,859 | | | 2,023 | 856.2 | | 25,859 | | 2,023 | |
|  | Survey preparation | | 159.1 | | 9,023 | | | 45,088 | 172.9 | | 10,496 | | 39,404 | |
|  | Training | | 1186.0 | | 55,692 | | | 10,539 | 1338.0 | | 59,181 | | 11,338 | |
|  | Survey execution | | 4675.2 | | 135,579 | | | 95,267 | 4532.0 | | 121,986 | | 95,267 | |
|  | Data entry | | 0.0 | | 0 | | | 0 | 1950.0 | | 43,535 | | 9,384 | |
|  | Data cleaning, processing, & preparation | | 535.5 | | 24,972 | | | 0 | 2159.5 | | 98,057 | | 0 | |
|  | Administration | | 353.5 | | 26,824 | | | 46,401 | 353.5 | | 26,824 | | 57,650 | |
|  | Grand total | | 7,765 | | 277,949 | | | 199,318 | 11,362 | | 385,937 | | 215,064 | |
|  | Number of respondents | | 4,376 | | 4,376 | | | 4,376 | 4,376 | | 4,376 | | 4,376 | |
|  | Total per respondent | | 2 | | 64 | | | 46 | 3 | | 88 | | 49 | |

24HR, 24hr dietary recall; INDDEX24, INDDEX24 Dietary Assessment Platform; PAPI, pen-and-paper interview; PSEM, portion size estimation method; USD, US dollars.

Supplemental Table S8. Time (human capital) and non-time (non-human capital) costs of conducting a national scale 24hr dietary recall using INDDEX24 and PAPI: Burkina Faso

|  | |  | | INDDEX24 | | | | | | PAPI | | | | |
| --- | --- | --- | --- | --- | --- | --- | --- | --- | --- | --- | --- | --- | --- | --- |
|  | |  | | Time | | Time cost | Non-time cost | | | Time | | Time cost | | Non-time cost |
| Primary activity | | Sub-activities | | (person days) | | (2019 USD) | (2019 USD) | | | (person days) | | (2019 USD) | | (2019 USD) |
| Preparation of dietary data inputs | Develop food and recipe lists and tags/probes | | 29.4 | | 2,957 | | | 0 | 29.4 | | 2,957 | | 0 | |
|  | Prepare food composition table | | 0.0 | | 0 | | | 0 | 0.0 | | 0 | | 0 | |
|  | Develop standard recipes density factors | | 97.8 | | 8,194 | | | 2,324 | 97.8 | | 8,194 | | 2,324 | |
|  | Identify PSEM/conversion factors | | 4.0 | | 704 | | | 2,188 | 4.0 | | 704 | | 2,188 | |
|  | Compile and format dietary reference data | | 13.1 | | 1,619 | | | 0 | 13.1 | | 1,619 | | 0 | |
|  | Sub-total | | 144.4 | | 13,474 | | | 4,512 | 144.4 | | 13,474 | | 4,512 | |
| Survey preparation | Design paper questionnaire for 24HR | | 0 | | 0 | | | 0 | 5.5 | | 963 | | 0 | |
|  | Develop data entry form for 24HR | | 0 | | 0 | | | 0 | 14.0 | | 657 | | 0 | |
|  | Pilot mobile app/paper questionnaires | | 105.9 | | 6,344 | | | 0 | 105.3 | | 6,240 | | 0 | |
|  | Develop manuals and training materials | | 1.0 | | 175 | | | 0 | 5.1 | | 893 | | 0 | |
|  | Translate forms and training materials | | 64.3 | | 5,825 | | | 0 | 63.9 | | 5,772 | | 0 | |
|  | Print survey instruments/questionnaires | | 20.0 | | 853 | | | 1,092 | 43.4 | | 4,943 | | 7,027 | |
|  | Print photo atlas | | 4.7 | | 820 | | | 5,986 | 4.7 | | 820 | | 5,986 | |
|  | Receive ethical approval | | 3.5 | | 170 | | | 1,841 | 3.5 | | 170 | | 1,841 | |
|  | Purchase and prepare supplies and equipment | | 56.3 | | 4,584 | | | 45,201 | 21.9 | | 1,816 | | 19,005 | |
|  | Purchase CommCare subscription | | 0.0 | | 0 | | | 5,000 | 0.0 | | 0 | | 0 | |
|  | Sub-total | | 255.6 | | 18,771 | | | 59,119 | 267.3 | | 22,273 | | 33,858 | |
| Training | Supervisor training | | 304.0 | | 20,838 | | | 6,368 | 304.0 | | 20,838 | | 6,368 | |
|  | Enumerator training | | 1313.0 | | 63,695 | | | 21,318 | 1313.0 | | 63,695 | | 21,318 | |
|  | Data entry clerk training | | 0.0 | | 0 | | | 0 | 222.0 | | 2,904 | | 459 | |
|  | Sub-total | | 1617.0 | | 84,533 | | | 27,685 | 1839.0 | | 87,436 | | 28,144 | |
| Survey execution | Household listing and sampling of eligible participants | | 2422.3 | | 121,007 | | | 43,100 | 2423.7 | | 121,011 | | 43,100 | |
|  | Incentives | | 0.0 | | 0 | | | 0 | 0.0 | | 0 | | 0 | |
|  | Data collection and field supervision | | 3241.3 | | 116,072 | | | 103,394 | 3241.3 | | 116,072 | | 103,394 | |
|  | Electronic data monitoring | | 285.2 | | 39,007 | | | 0 | 0.0 | | 0 | | 0 | |
|  | Sub-total | | 5948.8 | | 276,086 | | | 146,494 | 5664.9 | | 237,083 | | 146,494 | |
| Data entry | Data entry and supervision | | 0.0 | | 0 | | | 0 | 2860.0 | | 55,333 | | 2,563 | |
|  | Sub-total | | 0.0 | | 0 | | | 0 | 2860.0 | | 55,333 | | 2,563 | |
| Data cleaning, processing, and preparation | Data cleaning, processing (food matching, gap filling, etc.), and preparation for analysis | | 413.0 | | 49,207 | | | 0 | 1665.5 | | 193,126 | | 0 | |
|  | Sub-total | | 413.0 | | 49,207 | | | 0 | 1665.5 | | 193,126 | | 0 | |
| Administration | Management and oversight | | 306.2 | | 46,046 | | | 0 | 311.2 | | 47,100 | | 0 | |
|  | International travel to the field | | 0.0 | | 0 | | | 0 | 0.0 | | 0 | | 0 | |
|  | Lodging/per diem for international personnel | | 0.0 | | 0 | | | 0 | 0.0 | | 0 | | 0 | |
|  | Overhead | | 0.0 | | 0 | | | 73,736 | 0.0 | | 0 | | 88,178 | |
|  | Sub-total | | 306.2 | | 46,046 | | | 73,736 | 311.2 | | 47,100 | | 88,178 | |
| Totals | Prepare dietary reference data | | 144.4 | | 13,474 | | | 4,512 | 144.4 | | 13,474 | | 4,512 | |
|  | Survey preparation | | 255.6 | | 18,771 | | | 59,119 | 267.3 | | 22,273 | | 33,858 | |
|  | Training | | 1617.0 | | 84,533 | | | 27,685 | 1839.0 | | 87,436 | | 28,144 | |
|  | Survey execution | | 5948.8 | | 276,086 | | | 146,494 | 5664.9 | | 237,083 | | 146,494 | |
|  | Data entry | | 0.0 | | 0 | | | 0 | 2860.0 | | 55,333 | | 2,563 | |
|  | Data cleaning, processing, and preparation | | 413.0 | | 49,207 | | | 0 | 1665.5 | | 193,126 | | 0 | |
|  | Administration | | 306.2 | | 46,046 | | | 73,736 | 311.2 | | 47,100 | | 88,178 | |
|  | Grand total | | 8685.0 | | 488,116 | | | 311,547 | 12752.3 | | 655,826 | | 303,749 | |
|  | Number of respondents | | 6,500 | | 6,500 | | | 6,500 | 6,500 | | 6,500 | | 6,500 | |
|  | Total per respondent | | 1 | | 75 | | | 48 | 2 | | 101 | | 47 | |

24HR, 24hr dietary recall; INDDEX24, INDDEX24 Dietary Assessment Platform; PAPI, pen-and-paper interview; PSEM, portion size estimation method; USD, US dollars.
